# Gene-set Enrichment with Mathematical Biology (GEMB)

**DOI:** 10.1093/gigascience/giaa091

**Published:** 2020-10-09

**Authors:** Amy L Cochran, Kenneth J Nieser, Daniel B Forger, Sebastian Zöllner, Melvin G McInnis

**Affiliations:** Department of Math, University of Wisconsin–Madison, 480 Lincoln Drive, Madison, WI, 53706, USA; Department of Population Health Sciences, University of Wisconsin–Madison, 610 Walnut Street, Madison, WI, 53726, USA; Department of Population Health Sciences, University of Wisconsin–Madison, 610 Walnut Street, Madison, WI, 53726, USA; Department of Mathematics, University of Michigan, 530 Church Street, Ann Arbor, MI, 48109, USA; Department of Computational Medicine and Bioinformatics, University of Michigan, 100 Washtenaw Avenue, Ann Arbor, MI, 48109, USA; Department of Biostatistics, University of Michigan, 1415 Washington Heights, Ann Arbor, MI, 48109, USA; Department of Psychiatry, University of Michigan, 4250 Plymouth Road, Ann Arbor, MI, 48109, USA; Department of Psychiatry, University of Michigan, 4250 Plymouth Road, Ann Arbor, MI, 48109, USA

**Keywords:** mathematical biology, gene ontology, genetic enrichment, gene-set analysis, bipolar disorder, calcium signaling

## Abstract

**Background:**

Gene-set analyses measure the association between a disease of interest and a “set" of genes related to a biological pathway. These analyses often incorporate gene network properties to account for differential contributions of each gene. We extend this concept further—defining gene contributions based on biophysical properties—by leveraging mathematical models of biology to predict the effects of genetic perturbations on a particular downstream function.

**Results:**

We present a method that combines gene weights from model predictions and gene ranks from genome-wide association studies into a weighted gene-set test. We demonstrate in simulation how such a method can improve statistical power. To this effect, we identify a gene set, weighted by model-predicted contributions to intracellular calcium ion concentration, that is significantly related to bipolar disorder in a small dataset (*P* = 0.04; *n* = 544). We reproduce this finding using publicly available summary data from the Psychiatric Genomics Consortium (*P* = 1.7 × 10^−4^; *n* = 41,653). By contrast, an approach using a general calcium signaling pathway did not detect a significant association with bipolar disorder (*P* = 0.08). The weighted gene-set approach based on intracellular calcium ion concentration did not detect a significant relationship with schizophrenia (*P* = 0.09; *n* = 65,967) or major depression disorder (*P* = 0.30; n = 500,199).

**Conclusions:**

Together, these findings show how incorporating math biology into gene-set analyses might help to identify biological functions that underlie certain polygenic disorders.

## Background

Genetic contributions to disease can be complex and might involve the coordination of a collection of genetic variants in the disruption of 1 or many biological pathways. Previous studies of psychiatric conditions provide evidence that a single genetic variant often confers little disease risk despite high heritability [1–3]. Rather, psychiatric disorders can be polygenic [4]—hundreds to thousands of genes of very small effect contribute to the disorder. For this reason, genetic risk for an individual is commonly measured by aggregating information from multiple genes into a polygenic risk score [5–9]. Each of these variants might play a small role in the disruption of a pathway but collectively lead to the development of disease. Consequently, uncovering genetic influences on psychiatric disorders can be challenging when etiology of disease depends on >1 gene [10]. Computational approaches are emerging to better prioritize candidate genes [11–22].

Gene-set analyses are a common tool for measuring the association between a disorder and a set of genes rather than a single gene [23–28]. Many statistical tests and software are available to perform gene-set analysis (cf. [28, 29]) to determine whether genes in a particular gene set are significantly associated with a phenotype (self-contained) or whether a phenotype is more strongly associated with genes in a set than genes not in the set (competitive) [26, 28, 30]. Often gene sets are defined on the basis of genes that contribute to a particular biological pathway, which enables identification of pathways that are important for a disorder. This approach likely leads to stronger, more reproducible findings if abnormal pathways are what ultimately contributes to genetic risk [28, 31].

However, biological functions may ultimately drive risk as opposed to an abnormal pathway or single gene variant. Biological functions do not map one-to-one to biological pathways; a function can recruit some genes from multiple pathways [32]. In bipolar disorder, for example, spontaneous neuronal firing rate differs in stem cells derived from bipolar individuals compared with controls [33, 34]. This cellular function—neuronal firing rate—recruits genes from calcium-mediated signaling (GO:0019722), regulation of action potential (GO:0098900), and chemical synaptic transmission (GO:0007268), among others. Hence, if perturbed biological functions drive disease risk, jointly testing genes in 1 pathway that includes genes of little impact and ignoring genes in other relevant pathways would result in a less powerful gene-set analysis.

Moreover, some genes or gene products play a larger role in the realization of the biological function. To account for this, some gene-set analyses incorporate information about the network structure of gene interactions [12–15, 35–37]. Network-based methods, such as EnrichNet [13], GANPA [37], and LEGO [36], represent the network of functional interactions between genes as a network—a mathematical object made up of vertices (genes or proteins) and edges (connections). On the basis of features of the network, such as the number of connections a gene has with other genes, genes are given more or less weight in the gene-set test statistic. While these methods attempt to account for the functional non-equivalence of genes, they rely on mathematical properties of the network rather than biological mechanisms involved in the related biological function. The connections between genes might vary in strength or operate in a non-linear way. For example, a gene with many weak connections to other genes might carry equal or less downstream biological influence than a gene with few, strong connections to other genes. This discrepancy would be obscured by considering only the number or pattern of connections. Greater specificity can be achieved quickly through detailed mathematical models from math biology, which are driven from bottom-up biophysical principles. Efforts within the field have culminated in ModelDB [38], which hosts >1,000 publicly available models [39]. Examples include models of the hypothalamic-pituitary-adrenal axis, monoamine systems, and circadian rhythms, among others. Model parameters related to genes can be varied to measure the relative contribution of genes to a specific biological function of interest (e.g., firing rate). Incorporating model predictions into gene-set tests might strengthen the link between genes and disorders.

We present a simple method, Gene-set Enrichment with Math Biology (GEMB), for measuring the association between a disorder and genes connected to a biological function, based on model predictions. Our method relies on (i) ranking genes in decreasing order of association strength to a disorder and (ii) assigning weights to a set of genes to reflect their relative contribution to a specific biological function. We illustrate one approach to assigning weights by using pre-existing models from math biology. Ranks and weights are combined into a test for significance of the association between genes related to a biological function (as predicted by a neurobiological model) and a disorder.

To demonstrate the utility of our method, we test the hypothesis that genes affecting intracellular calcium ion (Ca^2+^) concentration are related to bipolar disorder by incorporating a detailed model of intracellular Ca^2+^ concentrations [40]. Bipolar disorder is a severe and chronic psychiatric disorder [41] with estimated heritability at 85% [42]. Genome-wide association studies report several susceptibility loci [43], including a voltage-gated calcium gene [44], which remains among the strongest findings to date. Calcium signaling is an incredibly complex process to model [45] but has been implicated in many human diseases [46], including bipolar disorder.

## Method Description

### A weighted gene-set statistic

**Figure 1:**
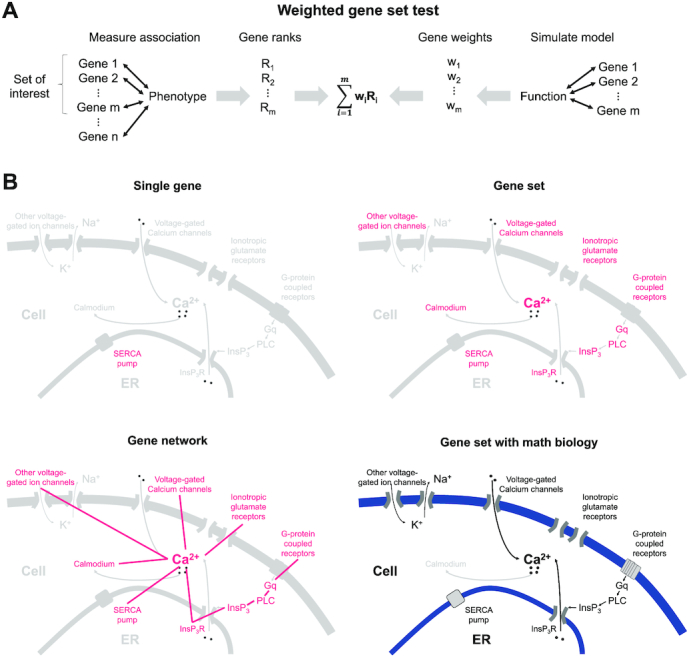
Overview of gene-set analysis with math biology. (A) Genes are ranked on the basis of their association with a phenotype and weighted on the basis of their model-predicted contribution to a specific function. Ranks and genes are combined to perform a weighted gene-set test. (b) Genetic analysis can be performed at the level of either a single gene, a gene set, a gene network, or a gene set connected by math biology. Gene-set analysis with math biology uses models to describe connections between genes based on biophysical principles.

We assume a general set-up of a competitive gene-set test: individuals are phenotyped and analyzed for expression in *n* genes; each gene is measured for association to the phenotype; and a subset of *m* genes are determined to be of interest (see Fig. 1 for an overview). From this set-up, we require only the rank of each gene in decreasing order of association strength to the phenotype; genes that are most strongly associated with the phenotype have the highest rank (i.e., closest to 1) and those that are most weakly associated with the phenotype have the lowest rank (i.e., closest to *n*).

We diverge from many gene-set tests by requiring that non-negative weights be assigned to individual genes in the subset of interest. Formally, we require:

- genes labeled 1 to *n*;
- rank *r_i_* ∈ {1, …, *n*} for each gene *i* = 1, …, *n*;
- gene set $\mathcal {S} \subseteq \lbrace 1, \ldots , n \rbrace$; and
- weights *w_i_* ≥ 0 for each gene $i \in \mathcal {S}$ with $\textstyle\sum _{i \in \mathcal {S}} w_i > 0$.

Without any loss of generality, we assume weights *w_i_* sum to 1; we can always rescale weights so that they sum to 1. Then, we define the following test statistic using a weighted sum of the ranks *r_i_* ($i \in \mathcal {S}$):

\begin{eqnarray*}
v := \textstyle\sum _{i \in \mathcal {S}} w_i r_i. \end{eqnarray*}

The choice of weights encodes an *a priori* hypothesis about the relative contribution of a gene to the phenotype. As a specific case, we can recover an unweighted gene-set test by setting *w_i_* = 1/*m*. This choice of weights captures the *a priori* hypothesis that each gene in $\mathcal {S}$ contributes equally to the phenotype (or a lack of support for one gene over another). In this case, the statistic *v* is the mean rank of the genes in $\mathcal {S}$. Recalling that a rank of 1 is assigned to the gene with the strongest association, a value *v* < (*n* + 1)/2 reflects that genes in $\mathcal {S}$ are ranked higher on average relative to genes not in $\mathcal {S}$. Conversely, a value *v* > (*n* + 1)/2 reflects that genes in $\mathcal {S}$ are ranked lower on average relative to genes not in $\mathcal {S}$. If *v* = (*n* + 1)/2, genes in $\mathcal {S}$ are ranked neither higher nor lower on average relative to genes not in $\mathcal {S}$. In other words, small *v* suggests an association between the gene set and phenotype. We point out that genes in $\mathcal {S}$ do not need to be evenly distributed in rank to achieve *v* ≈ (*n* + 1)/2; they could be disproportionately ranked close to the mean rank (*n* + 1)/2 or ranked close to the extreme ranks 1 and *n*.

As another specific case, we could recover a single gene test by setting *w_j_* = 1 for some $j \in \mathcal {S}$ and setting all other weights to zero. This choice captures the *a priori* hypothesis that gene *i* specifically contributes to the phenotype. The statistic *v* would be the rank of gene *i*. More broadly, setting any weight to zero reflects the hypothesis that the corresponding gene does not contribute to the phenotype. The statistic *v* would be identical in value if we had simply removed the gene from $\mathcal {S}$. Further, smaller weights mean a smaller contribution to *v*.

With more general weights, the statistic *v* is interpreted similarly to the unweighted version, replacing an average of the ranks with a weighted average. Our interpretation is inherited from the fact that *v* − (*n* + 1)/2 changes sign when genes are ranked in opposite order and increases when a gene in $\mathcal {S}$ is exchanged for a gene not in $\mathcal {S}$ with higher rank. Thus, small *v* can be thought of as showing that genes in $\mathcal {S}$ have a higher (weighted) relative rank to genes not in $\mathcal {S}$.

### A weighted gene-set test

To use *v* in a statistical test, we must specify a null distribution. For many gene-set analyses, a common null hypothesis is that the genes in $\mathcal {S}$were chosen uniformly at random from the entire set of genes. Under this null hypothesis, we can construct a null distribution for *v* by drawing ranks for genes in our set, *R_i_* for $i \in \mathcal {S}$, uniformly at random from

\begin{eqnarray*}
\lbrace 1, 2, \ldots , n\rbrace
\end{eqnarray*}

without replacement and calculating

\begin{eqnarray*}
V := \textstyle\sum _{i \in \mathcal {S}} w_i R_i. \end{eqnarray*}

The distribution of the random variable *V* serves as the null distribution for *v*.

The alternative hypothesis is that genes in $\mathcal {S}$ were not chosen uniformly at random. In broad terms, they were chosen because of their relationship to the phenotype. Hence, we are interested in how often *V* with gene ranks chosen randomly suggests a stronger association between set $\mathcal {S}$and a phenotype than the statistic *v* determined by the actual association to the phenotype. In other words, we use the probability, or *P*-value, associated with a 1-sided test given by

\begin{eqnarray*}
\mathbb {P}\left( V \le v \right) \end{eqnarray*}

to determine whether *v* is significant. Note, a 2-sided test could also be defined by using

\begin{eqnarray*}
\mathbb {P}\left( \left| V - \frac{n+1}{2} \right|\ge \left| v - \frac{n+1}{2} \right| \right). \end{eqnarray*}

A simple way to estimate $\mathbb {P}\left( V \le v\right)$ is to use Monte Carlo simulation, where *V* is repeatedly sampled from its distribution and we count how often a sample of *V* ≤*v*. This computation benefits from the fact that *V* is simple to calculate and can be sampled in parallel. The law of large numbers ensures that a Monte Carlo estimate of $\mathbb {P}\left( V \le v \right) = \mathbb {E}( 1_{V \le v} )$ is unbiased and has variance $\mathrm{Pr} ( V \le v )/k$, where *k* is the number of Monte Carlo samples.

#### Asymptotic approximation

Alternatively, we could estimate $\mathbb {P}( V \le v )$ with

\begin{eqnarray*}
\Phi \left( \frac{v-\mu }{\sigma _w} \right), \end{eqnarray*}

where φ is a standard normal distribution and μ = (*n* + 1)/2 and $\sigma _w^2 = \left[ \left( n^2-1 \right)/12 \right] \textstyle\sum _{i\in \mathcal {S}} w_i^2$. This approximation follows by making the simplifying assumption that ranks *R_i_* are drawn uniformly at random from {1, …, *n*} with replacement (as opposed to without replacement) and then noting that the resulting *V* is a sum of independent random variables with respective means *w_i_*[(*n* + 1)/2] and variances $w_i^2 \left[ \left(n^2-1 \right)/12 \right]$($i \in \mathcal {S}$). Table 1 compares Monte Carlo estimates of 1-sided *P*-values to estimates using a normal approximation.

**Table 1:** Difference between Monte Carlo estimates of a 1-sided *P*-value $\mathbb {P}(V \le v)$ for the weighted gene-set test and estimates using a normal approximation

*l*	*m*	*n*	*v*			
			μ − 4σ_*w*_	μ − 3σ_*w*_	μ − 2σ_*w*_	μ − σ_*w*_
0.5	10	1,000	−3.0E−05	−5.7E−04	−1.2E−03	2.2E−03
		10,000	−2.9E−05	−5.3E−04	−8.0E−04	3.0E−03
	100	1,000	−2.1E−05	−5.5E−04	−4.7E−03	−1.1E−02
		10,000	−8.0E−06	−1.2E−04	−6.1E−04	−7.2E−04
1	10	1,000	−3.1E−05	−7.4E−04	−1.5E−03	3.8E−03
		10,000	−3.1E−05	−7.0E−04	−1.1E−03	4.4E−03
	100	1,000	−1.8E−05	−5.0E−04	−4.0E−03	−8.7E−03
		10,000	−9.2E−06	−9.9E−05	−5.1E−04	−5.6E−04
2	10	1,000	−3.2E−05	−1.1E−03	−2.4E−03	6.7E−03
		10,000	−3.2E−05	−1.1E−03	−2.1E−03	7.0E−03
	100	1,000	−1.7E−05	−4.4E−04	−3.1E−03	−6.2E−03
		10,000	−8.2E−06	−1.6E−04	−4.0E−04	−7.3E−05

Weights were defined as *w_i_* ∝ *i*^*l*^ (*i* = 1, …, *m*) for various *l*, assuming *s* = {1, …, *m*}. A total of 10^7^ Monte Carlo samples were used in each case.

#### Type I error and power

Type I error is controlled by the distribution of gene ranks under the null hypothesis of no association between the gene set and the phenotype. Our weighted gene-set test uses the null distribution that arises when any permutation of gene ranks is equally likely. However, the true distribution of gene ranks when there is no association is not clearly defined owing to the complex correlations that might exist among genes. Moreover, the null distribution of gene ranks is determined by the method used to measure gene–phenotype associations (see [47] for a comparison). It is thus important to choose a method for ranking genes that properly controls Type I error.

**Figure 2:**
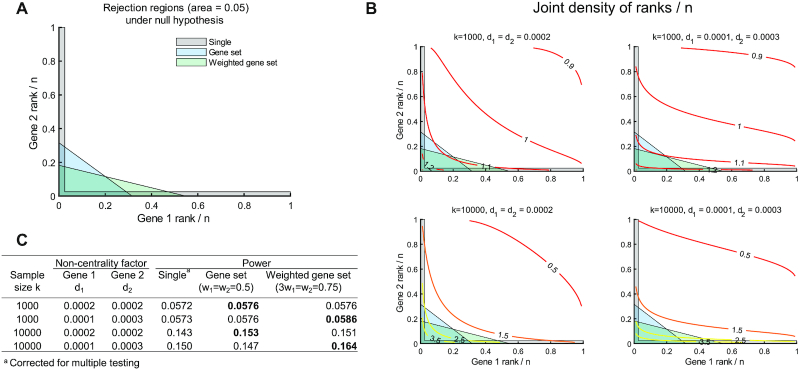
Statistical power. (A) Possible regions to reject null hypothesis for a single-gene test (corrected for multiple testing), a gene-set test, and a weighted gene-set test. (B) Joint density functions of ranks divided by *n* for different sample sizes (*n*) when genes have very small effect sizes (*d*_1_ and *d*_2_). (C) Statistical power estimated for each case in (B). The larger power between methods (gene set versus weighted gene set) is bolded in each case.

Power can be improved with a weighted gene-set test over gene-set or single-gene analyses when multiple genes have differential contribution to disease risk. To illustrate, consider *n* genes and a set of 2 independent genes with very small association to the disease. Under our null hypothesis, gene ranks divided by *n* are approximately uniformly distributed between 0 and 1. A single-gene test could assess whether each gene's normalized rank is below some critical value (Fig. 2A, gray region). By contrast, a gene-set test could assess whether the sum of the 2 genes' normalized ranks is below some threshold (Fig. 2A, blue region) and a weighted gene-set test could assess whether a weighted sum of the 2 genes' normalized ranks is below some threshold (Fig. 2A, green region). In each case, Type I error is controlled at 0.05 when the rejection region has an area of 0.05.

To estimate statistical power, we consider a situation when 2 independent genes of interest are ranked on the basis of an F test examining whether *ν* coefficients are zero when regressing phenotype on gene variables, as is done in MAGMA, with *ν* being the number of gene-level principal components used in the regression model [26]. For simplicity, we set *ν* = 10 and assume that the *P*-value for each gene recovered from the F test would be its rank normalized by the number of genes *n*. For a sample size of *k*, the test statistic for gene 1 and 2 would follow an F distribution with *ν* − 1 and *k* − *ν* degrees of freedom under the null hypothesis (no gene–phenotype association). For an alternative distribution, we assume that the test statistic follows a non-central F distribution with *ν* − 1 and *k* − *ν* degrees of freedom and non-centrality parameters *kd*_1_ or *kd*_2_ for gene 1 and 2, respectively. Under this alternative, increasing sample size or non-centrality leads to larger joint densities for normalized ranks near the axes (Fig. 2B). Thus with only 2 genes, these changes can improve statistical power—the probability of arriving at normalized ranks that lie in each reject region (Fig. 2C). We expect that this improvement would continue to hold or grow with increases in gene-set size and increasingly differential effect sizes. Hence, this example provides support that weighting normalized gene ranks can further increase statistical power by accounting for differential contributions of genes to a disease.

#### Gene correlation

As alluded to above, nearby genes are often correlated. This correlation could lead to correlated gene ranks and subsequently violate the null distribution arising from drawing gene ranks uniformly at random without replacement. To account for gene correlation, we can draw ranks distributed as a multivariate normal random variable (ϵ_1_, …, ϵ_*n*_) with mean zero and covariance matrix Σ and let *R_i_* be the rank of ϵ_*i*_ among the set {ϵ_1_, …, ϵ_*n*_}. The covariance matrix Σ captures gene correlation. The resulting distribution for $V=\textstyle\sum _{i \in \mathcal {S}} w_i R_i$can then be estimated by Monte Carlo simulation as before.

### Determining gene weights

Weights can capture any *a priori* hypothesis whether justified by functional data, literature surveys, or experiments. Our goal, however, is more specific: we want weights to reflect the relative contribution of genes to a specific biological function. If we have reason to think certain genes play a large role in the biological function of interest, we upweight them. If genes do not affect the biological function of interest, we downweight them. In this way, our weighted gene-set test incorporates the hypothesis that a specific biological function (captured by the weights) is important to a phenotype.

To inform the choice of weights, we propose a general approach using models from math biology. We start with a neurobiological model that can return a scalar measure of the function of interest. As noted earlier, many models are publicly available through sources such as ModelDB [38]. Next, we consult gene databases to identify genes related to 1 or more model parameters and create a mapping of genes to model parameters. Then, we perform a global sensitivity analysis to measure the relative contribution of each parameter to a specific function of interest. We opt for a global sensitivity analysis based on the partial rank correlation coefficient (PRCC) [48] due to its simplicity. Last, we assign weights to each gene based on the contributions of the model parameter to which it is mapped.

We remark that the association between genes and parameters need not be one-to-one. On one hand, models might not be sufficiently detailed to capture the individual contribution of each gene, so multiple genes may be associated with a single parameter. For example, 4 genes are known to modulate formation of L-type Ca^2+^ channels, but most mathematical models with L-type ion channels do not include individual parameters to capture the differential contributions of each gene. On the other hand, multiple parameters might be associated with a single gene. For example, models of neuronal action potential often distinguish between sodium currents and persistent sodium currents [49] even though both currents may be regulated by the same gene [50]. We describe how we handled these issues in the context of our case study.

## Analyses

To illustrate our method, we explore the hypothesis that genes contributing to intracellular Ca^2+^ concentrations in excitable neurons are related to bipolar disorder. Calcium signaling has been both implicated in bipolar disorder and extensively modeled. Furthermore, this hypothesis was initially tested using our method with a relatively small dataset (*n* = 544) from the Prechter Bipolar Cohort [51] (details in Appendix). Thus, the results reported here reproduce our initial finding and validate an *a priori* hypothesis with a much larger dataset.

### Gene ranks

Summary genetic data were obtained on patients with bipolar disorder (*n* = 20,129) and controls (*n* = 21,524) from the Psychiatric Genomics Consortium (PGC) [52, 53]. Association was measured between 8,958,989 SNPs and bipolar disorder, resulting in *P*-values for each SNP. Data collection and analysis are detailed in Ruderfer et al. [53]. Using SNP-level summary data, gene-level association with bipolar disorder was measured using MAGMA software [26]. Default parameter settings were used in MAGMA, with gene boundaries defined on the basis of NCBI Build 37 (hg19). A total of 3,554,879 (39.68%) SNPs mapped to ≥1 gene, whereas 18,309 genes (out of 19,427 genes) mapped to ≥1 SNP. Linkage disequilibrium between SNPs was estimated by MAGMA using reference data files created from Phase 3 of 1,000 Genomes [54]. The set of 18,309 genes were ranked on the basis of their measured association (*P*-value) with bipolar disorder; the smallest *P*-values were ranked closest to 1.

### Gene weights

We used a detailed model of an intracellular Ca^2+^ concentration in a hippocampus CA1 pyramidal cell developed by Ashhad and Narayanan [40]. The model is publicly available in ModelDB (Model 150551) and written with free Neuron software [38]. Furthermore, it captures key contributors to intracellular Ca^2+^ concentrations, including ion transport (K^+^, Na^+^, and Ca^2+^) across the cell membrane; transport of Ca^2+^ into and out of the sarcoplasmic endoplasmic reticulum; synaptic plasticity; and mediating receptors such as inositol triphosphate (InsP_3_), ionotropic glutamate receptors, and metabotropic glutamate recptors. Finally, the model uses a morphologically realistic 3D representation of a hippocampus CA1 pyramidal cell accompanied by spatial dynamics giving rise to Ca^2+^ waves.

To identify genes of interest, we started with 182 genes making up the “Calcium signaling pathway" (Pathway ko04020) in KEGG [55–57]. Each gene was evaluated for whether it could modulate intracellular Ca^2+^ concentrations in the model using the Gene database from the NCBI [58]. A total of 38 genes could modulate intracellular Ca^2+^ concentrations in the model, by way of ion channels, ion pumps, or receptors. We found 3 ion channels (Na^+^, A-type K^+^, and delayed rectifying K^+^) and 2 receptors (N-methyl-D-aspartate [NMDA] and α-amino-3-hydroxy-5-methyl-4-isoxazolepropionic acid [AMPA]) that could affect intracellular Ca^2+^ concentration in the model but had not been associated with genes in the KEGG calcium signaling pathway. An additional 31 genes were found related to these channels or receptors. Of the 69 genes identified, 4 genes (*ATP2B3, CACNA1F, GRIA3, KCND1*) were excluded because they were not associated with gene ranks (described below). A total of 65 genes were analyzed.

For each gene, we identified a parameter that could modulate (up and down) the modeling component related to the gene. For example, channel conductance was associated with ion channel genes. Default parameter values were taken from the simulation in Fig. 6 of [40]. Other genes, associated parameters, and default values are summarized in Table 2.

**Table 2:** Calcium genes and associated model parameters

Genes	Value	Parameter
*ATP2B[1-2,4]*	0.008 μM ms^−1^	Mean rate γ_0_ of Ca^2+^ flux density
*ATP2A[1–3]*	0.1 μM ms^−1^	Amplitude *V*_max_ of SERCA pump uptake
*CACNA1[C–D,S]*	0.316 mS cm^−1^	L-Type Ca^2+^ channel conductance *g*_CaL_
*CACNA1[G–I]*	0.1 mS cm^−1^	T-Type Ca^2+^ channel conductance *g*_CaT_
*GRM[1,5]*	0.3e-3	Metabotropic glutamate receptor density [mGR]_0_
*GNA[Q,11,14–15]*	100 ms^−1^	G_α_-bound activated PLC formation rate k_7_
*PLC[B1–B4,D1,D3–D4,E1,G1–G2,Z1]*	0.83 ms^−1^	PLC_α_-bound PIP_2_ formation rate k_9_
*ITPR[1–3]*	1.85	IP_3_ receptor density $\mathrm{\bar{g}_{InsP_3R}}$
*GRIN[1,2A–2D,3A–3B]*	1.938107025 nM s^−1^	Maximum NMDA receptor permeability $\mathrm{\bar{P}_{NMDA}}$
*GRIA[1–2,4]*	1.29207135 nM s^−1^	Maximum AMPA receptor permeability $\mathrm{\bar{P}_{AMPA}}$
*KCN[A4,C3–C4,D2–D3]*	22 mS cm^−2^	A-type K^+^ channel conductance *g*_KA_
*KCN[A1–A3,A6–A7,B1–B2,C1–C2]*	3 mS cm^−2^	Delayed rectifying K^+^ channel conductance $\mathrm{\bar{g}_{KDR}}$
*SCN[1–5,8–11]A*	90 mS cm^−2^	Na^+^ channel conductance $\mathrm{\bar{g}_{Na}}$

Calcium genes affect either ion channels, ion pumps, or receptors in the Ashhad and Narayanan model [40]. Baseline parameter values were taken from [40].

With parameters and genes identified, we used the Ashhad and Narayanan model to simulate intracellular Ca^2+^ concentrations during an established protocol for inducing synaptic plasticity at a synapse, namely, 900 pulse stimulation at 10 Hz; see Fig. 6 in [40]. We simulated 320 samples of parameter sets using Latin-hypercube sampling from a normal distribution with mean given by the respective baseline parameter in [40], standard deviation given by 5% of the respective baseline parameter, and zero correlation. For each parameter set, we simulated intracellular Ca^2+^ and measured average intracellular Ca^2+^ concentrations during initial transients induced in the first 3 seconds of the simulation.

**Figure 3:**
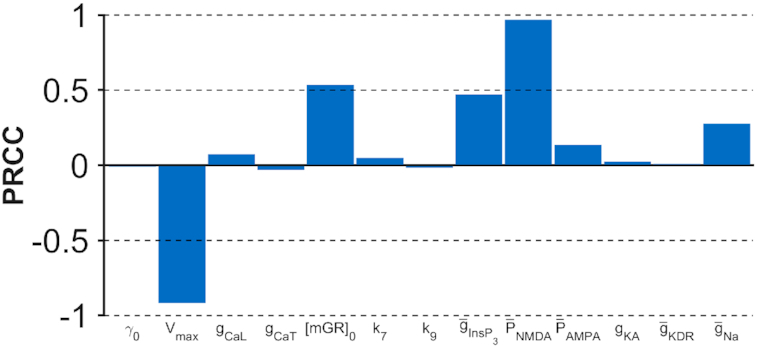
Partial rank correlation coefficient (PRCC) estimated for 13 parameters that modulate intracellular Ca^2+^ concentrations in the Ashhad and Narayanan model [40]. PRCC measures partial correlation between a parameter and the functional measure of interest (mean intracellular Ca^2+^ concentration) controlling for the contribution of other parameters.

We estimated the PRCC between each parameter and the measured concentrations controlling for the remaining parameters (Fig. 3). We found, for example, a strong positive partial correlation between mean intracellular Ca^2+^ concentrations and maximum permeability $\mathrm{\bar{P}_{NMDA}}$ of NMDA receptors and a strong negative partial correlation between mean intracellular Ca^2+^ concentrations and the amplitude *V*_max_ of sarcoplasmic/endoplasmic reticulum calcium (SERCA) pump uptake.

Based on estimated PRCCs, we defined weights for the 65 genes as follows. For each of the *N_k_* genes assigned to parameter *k* with PRCC ρ_*k*_, we assigned weights |ρ_*k*_|/*N_k_*. We then renormalized weights to sum to 1. Note that we could use any function of ρ_*k*_ to assign weights to associated genes. We use only the magnitude of PRCC because measured associations between genes and phenotypes are not sufficiently specific to reflect the direction of association in addition to the magnitude. We divide by the number of genes assigned to parameter *k*, so that a single component in the model is not weighted heavily simply because there are a large number of genes assigned to the component.

### Weighted gene-set test

Combining gene ranks obtained from the genetic analysis with gene weights obtained from the model of calcium signaling, we performed the weighted gene-set test. For comparison, we performed an unweighted gene-set test using all 182 genes from the KEGG calcium signaling pathway [55–57] by assigning equal weights to all 182 genes. In addition, we performed a typical over-representation analysis with the set of 182 genes. Genes were labeled as significant or not based on a significance level of 0.1 adjusted for false discovery rate [59] (a significance level 0.0044 for our problem); a 1-sided Fisher exact test was performed to test for over-representation of significant genes in the KEGG calcium signaling pathway compared to genes not in the KEGG calcium signaling pathway.

**Figure 4:**
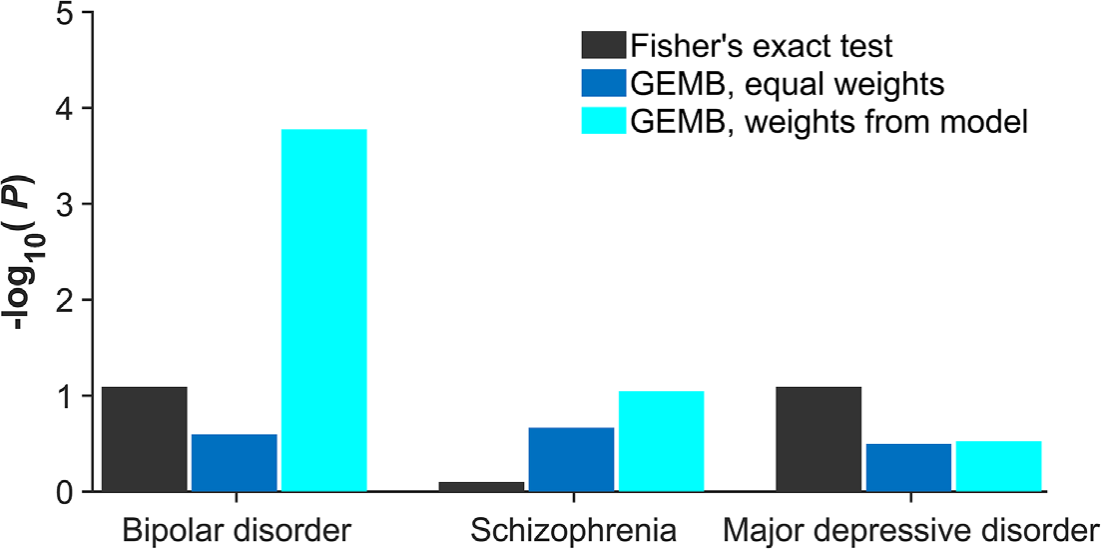
One-sided *P*-values estimated for gene-set tests. Three tests were performed for each disorder: (i) over-representation test (Fisher exact test) applied to the KEGG calcium signaling pathway, (ii) our gene-set test (GEMB) with equal weights applied to the entire KEGG calcium signaling pathway, and (iii) our gene-set test (GEMB) with genes related to the Ashhad and Narayanan model [40] and weighted according to their relative contribution to our functional measure of interest (mean intracellular Ca^2+^ concentration in an excitable cell).

Our gene-set test (GEMB) showed strong support for our hypothesis that genes contributing to intracellular Ca^2+^ concentration are related to bipolar disorder (*P* = 1.7 × 10^−4^; Fig. 4). Furthermore, focusing on the entire KEGG calcium signaling pathway without consideration of differential contributions to biological function provided little support for the hypothesis that calcium signaling is important to bipolar disorder (*P* = 0.26 using our method GEMB with equal weights and *P* = 0.081 using a 1-sided Fisher exact test). These discrepancies show that incorporating weights could possibly be illuminating biological factors that contribute to a psychiatric disorder.

### Sensitivity analyses

Additional analyses were performed to evaluate the sensitivity of our result to 4 factors. First, we wanted to ensure that our test would not simply find intracellular Ca^2+^ concentration to be important for any disease because such lack of specificity would limit the practical value of our method. An identical procedure used for the bipolar disorder dataset was applied to datasets for schizophrenia (33,426 cases and 32,541 controls) [53] and major depressive disorder (170,756 cases and 329,443 controls) [60] using summary data from the PGC (Fig. 4). Even though these 2 disorders have shared genetic risk with bipolar disorder, our weighted gene-set test did not find evidence to support the hypothesis that genes contributed to intracellular Ca^2+^ concentration are related to schizophrenia (*P* = 0.09) or major depressive disorder (*P* = 0.30). Neither our method with equal weights nor a 1-sided Fisher exact test suggested that the entire KEGG calcium signaling pathway was significantly related to schizophrenia or major depressive disorder (*P* > 0.05). Thus, our weighted gene-set test supports that these findings are specific to bipolar disorder.

Second, we wanted to ensure that our result was not driven by a single gene. We performed the weighted gene-set test repeatedly, removing each gene 1 at a time and recovering a *P*-value for each test. Regardless of which gene was removed, our test still found that the weighted gene set related to intracellular Ca^2+^ concentration was significantly associated with bipolar disorder. Through this process, we identified a list of 10 genes that contributed the most evidence—based on the largest increases in the *P*-value recovered after removing the given gene—to the association between the weighted gene set and bipolar disorder (Table 3). The top 10 genes are involved in SERCA pumps, inositol 1,4,5-trisphosphate (IP_3_) receptors, and ionotropic and metabotropic glutamate receptors. We also verified that our result was not driven by the *CACNA1C* gene, which is important because motivation for studying calcium signaling was driven in part by prior PGC results that implicate *CACNA1C*. The *CANA1C*gene alone was significant, ranking 10th out of 18,195 genes (*P* = 10/18,195 = 5.5 × 10^−4^), but our gene-set test continued to provide strong support for the remainder of the gene set contributing to intracellular Ca^2+^ with the *CACNA1C* gene removed (*P* = 1.9 × 10^−4^).

**Table 3:** Top 10 genes contributing to statistical significance of intracellular Ca^2+^ concentrations

Gene	Functional target	*P*
*ATP2A1*	Sarcoplasmic/endoplasmic reticulum calcium ATPase (SERCA)	1.2 × 10^−3^
*ITPR3*	Inositol 1,4,5-trisphosphate (IP_3_) receptor	1.1 × 10^−3^
*ATP2A2*	Sarcoplasmic/endoplasmic reticulum calcium ATPase (SERCA)	8.1 × 10^−4^
*GRIN2A*	Ionotropic glutamate receptor (NMDA)	6.6 × 10^−4^
*GRM1*	Metabotropic glutamate receptor	4.9 × 10^−4^
*GRIN2B*	Ionotropic glutamate receptor (NMDA)	4.4 × 10^−4^
*GRM5*	Metabotropic glutamate receptor	4.2 × 10^−4^
*GRIN3B*	Ionotropic glutamate receptor (NMDA)	2.7 × 10^−4^
*GRIA4*	Ionotropic glutamate receptor (AMPA)	2.4 × 10^−4^
*SCN2A*	Voltage-gated sodium channel	2.1 × 10^−4^
Baseline		1.7 × 10^−4^

Genes are ranked in order of largest *P* value after applying our gene-set test with the gene removed.

Third, we checked the sensitivity of our result to gene boundaries as defined by NCBI Build 38 because SNPs outside the gene boundary may still be relevant to the gene. We generated new gene ranks using MAGMA but extended the gene boundary by 10 kb in either direction. With these gene ranks, our test still found the weighted gene set contributing to intracellular Ca^2+^ concentration to be significantly related to bipolar disorder (*P* = 9.6 × 10^−4^), albeit to a lesser extent.

Last, we checked the sensitivity of our result to correlation between genes. An estimated gene correlation matrix was recovered from MAGMA software. Because the resulting matrix was not positive definite, we adjusted the smallest eigenvalues to be at least a value of 10^−6^, leaving the eigenvectors alone. The adjusted gene matrix Σ was then incorporated into the weighted gene-set test to account for gene correlation as described above. When accounting for gene correlation, our weighted gene-set test still found that intracellular Ca^2+^ concentration was related to bipolar disorder (*P* = 1.7 × 10^−4^).

## Discussion

We presented a method for examining associations between biological functions and psychiatric disorders that we call GEMB (Gene-set Enrichment with Math Biology). Central to our method are gene weights that measure the relative contribution of a gene to a particular biological function, which we determine using a neurobiological model. We applied our approach to assess the hypothesis that genes involved in the regulation of intracellular Ca^2+^ concentrations are related to bipolar disorder. Gene weights were based on their relative contribution to intracellular Ca^2+^ concentrations, determined by a detailed model of calcium signaling from Ashhad and Narayanan [40]. Gene ranks were obtained using summary genetic data from the PGC on bipolar disorder [53], consisting of 20,129 individuals with bipolar disorder and 21,524 controls. Combining gene ranks and weights with our weighted gene-set test, we found strong support for the hypothesis that the gene set contributing to intracellular Ca^2+^ concentrations is related to bipolar disorder (*P* = 1.7 × 10^−4^) compared to little support based on a test using the more general KEGG calcium signaling pathway (*P* = 0.081). This result illustrates how gene sets defined on the basis of biological pathways may be too broad to capture the genetic effect on a biological *f*unction that is associated with a disorder.

A practical benefit of our weighted gene-set test is that only gene ranks are needed from genetic data. Gene ranks can be shared across researchers more easily and require fewer regulatory and computational resources to analyze compared with full genetic data. Sharing genetic resources and data has become the norm in genetic research as the community moves towards large consortia to achieve the sample sizes, level of evidence, and study consistency that are expected. The PGC, for example, has ~300 investigators and >75,000 subjects [61], and the National Institute of Mental Health (US) has made genetic data available to researchers. Gene ranks can even be recovered from summary data rather than full genetic data as done by MAGMA [26].

Popular gene-set tests typically start with gene-level measures of association between genes and the phenotype such as fold change, *t*-statistic, *P*-value, gene ranks, likelihood ratio, regression coefficient, and correlation coefficient [25, 30, 62, 63]. We transformed *P*-values obtained from MAGMA into gene ranks. The choice of gene-level measure and any subsequent transformation is an important decision, because it can alter the findings from gene-set analyses in several ways. First, the gene-level measure may yield better accuracy in finding significant gene sets depending on the genetic architecture of the disease of interest [62, 64]. A binary variable may be better than a gene rank for diseases with a few highly influential genes but worse for diseases with a large number of moderately influential genes [64]. Second, gene-level measures differ in their statistical properties. For example, larger sample sizes may change *P-*values without affecting gene ranks, and using a probit transformation of *P*-values rather than *P*-values directly, as in MAGMA, can yield a distribution closer to normal [26]. Third, the choice of statistic could change the clinical relevance of the findings, as one finds in a single-gene analysis when comparing fold change to a *t*-statistic [65]. Although we use gene ranks, ranks can be easily replaced by any gene-level measure of association if desired. It will be important for future studies to explore how different gene-level measures fare with the weighted gene-set test that we propose here.

Once gene ranks are determined, our method then needs only gene weights from neurobiological models, which too has its benefits. Neurobiological models are numerous, experimentally validated, and publicly available in ModelDB [38]. For example, we were able to quickly explore calcium signaling in bipolar disorder, owing to the accessibility of a detailed model developed by Ashhad and Narayanan [40] (Model 150551). Similar quick explorations could be used to examine other potentially important biological functions. In searching key words in ModelDB, we found 171 models that contain the concept of “synaptic plasticity," 168 models that contain the concept of “calcium dynamics," 47 models that contain the dopamine neurotransmitter, and 9 models that contain the concept of “circadian rhythms," to name a few [38]. Together, these models could annotate genes on the basis of model-predicted functional measures to add to current resources that annotate genes on the basis of biological pathways, such as KEGG [55–57].

With GEMB, neurobiological models may inform genetic studies, but the reverse may also be true: genetic studies may inform neurobiological models. In psychiatry, for instance, there is growing emphasis on team science, affording many opportunities for researchers from the mathematical sciences to help tackle problems [66]. However, just as it is difficult to pin down genes to study in psychiatric disorders, it is also difficult to pin down specific biological processes to study because abnormal function is found for many neural systems in a psychiatric disorder [67]. Thus, GEMB could help identify, or ground, candidate neurobiological models for studying in psychiatry. The model of Ashhad and Narayanan [40] provides 1 such example.

There are a number of other methods like GEMB that try to incorporate information about gene function into gene-set analyses. Network-based methods represent genes as vertices and interactions between 2 genes as edges, resulting in a mathematical object known as a network [12–18, 35–37]. Properties of this network (e.g., their location in the network, number of connections to a gene) are used to adjust the weight given to a gene in a gene-set analysis. In a broad sense, genes might be important to a biological pathway if they are more connected to other genes. For example, GANPA [37] and LEGO [36] builds a network based on a gene co-expression, protein-protein interactions, and gene ontology. Based on this network, they weight each gene in the set of interest as a function of the number of connections to other genes in the set and other genes in the entire network. EnrichNet uses a random walk on these networks to measure associations between genes and target cellular processes. Alternative ways to incorporate functional information about genes include Bayesian approaches that account for overlap between gene sets [19, 20] or approaches based on gene expression levels [21, 22]. A benefit of our method is that it is sufficiently general, such that weights could also be determined from network analysis, experiments, or meta-analysis. Weights need only be non-negative and sum to 1.

The presented method GEMB was designed to be simple, which has certain limitations. First and foremost, our method like many genetic analyses ignores the various ways that gene and SNP interactions can influence a disease, resulting in a complex genetic architecture of the disease. For example, 1 gene or part of a gene can regulate another gene, or 2 genetic variants may lead to increased risk in a disorder that surpasses the additive risk of each variant alone. Hence, our method like other gene-set analyses aims to identify genes that are associated with disorders for further scientific investigation rather than to establish causal relationships between genes and disorders. Second, we do not account for gene interactions in the neurobiological model. The Sobol method of global sensitivity analysis [68], for example, could measure the relative contribution of parameters and their higher-order interactions. Our weighted gene-set test could be extended to incorporate these interactions. Third, neurobiological models are sure to be imperfect, meaning that gene weights are only predicted measures of biological function. This issue is, of course, common to all modeling. The question then is not whether using a model leads to the correct answer but rather whether using models to favor certain genes would strengthen inferences compared to treating the genes equally. This is an empirical question that only continued analyses and applications can answer.

In summary, we propose an approach to gene-set analysis that incorporates math biology. Our method can be used flexibly, requiring only that genes be ranked and weighted. Genes can be ranked using any algorithm, even when only summary genetic data are available. Ranks can be determined for any disease. Genes can be weighted using any information whether from experiments, prior analyses, simulation, or math biology—although we focused on the latter. Weights can even be reused from one disease to the next. When the underlying model of math biology is complicated, a researcher could use their own knowledge or borrow weights from another study (e.g., weights for intracellular Ca^2+^ concentration from the present study). These features, together with increasing availability of genetic datasets and models, leave few barriers to our method's use. In turn, our method may help to improve statistical power in gene-set analyses. Most importantly, it could facilitate meaningful biological interpretations that are ultimately necessary in our understanding of the genetic basis of disease.

## Availability of Source Code and Requirements

- Project name: GEMB
- Project home page: https://github.com/cochran4/GEMB
- RRID:SCR_018904
- biotoolsID: gemb
- Operating system(s): Platform independent
- Programming language: MATLAB
- Other requirements: None
- License: GNU GPL

## Availability of Supporting Data and Materials

The datasets supporting the results of this article are based on published work on schizophrenia and bipolar disorder as part of the PRC; data are available through the PGC website [69].

An archival copy of the source code and supporting data is available via the *GigaScience* database GigaDB [70].

## Abbreviations

AMPA: α-amino-3-hydroxy-5-methyl-4-isoxazolepropionic acid; GEMB: Gene-set Enrichment with Math Biology; IP_3_: inositol 1,4,5-trisphosphate; kb: kilobase pairs; KEGG: Kyoto Encyclopedia of Genes and Genomes; NCBI: National Center for Biotechnology Information; NMDA: N-methyl-D-aspartate; PGC: Psychiatric Genomics Consortium; PRCC: partial rank correlation coefficient; SERCA: sarcoplasmic/endoplasmic reticulum calcium; SNP: single-nucleotide polymorphism.

## Ethical Approval

For results reported in the Appendix, the University of Michigan's Biomedical Institutional Review Board approved all recruitment, assessment, and research procedures (HUM606). Patients provided written informed consent after receiving a complete description of the study.

## Competing Interests

M.G.M. has consulted with and/or received grant funding from Janssen Pharmaceuticals and Takeda Pharmaceuticals; he is a co-owner in Priori-AI, LLC. D.B.F. is the CSO of Arcascope and has equity in the company. Arcascope did not sponsor this research. All other authors declare that they have no competing interests.

## Funding

This research is supported by the Heinz C Prechter Bipolar Research Fund, the Richard Tam Foundation, a Human Frontiers of Science Program Grant (RPG 24/2012), the National Science Foundation (US; DMS grant 1714094), and the National Institute of Mental Health (US; K01-MH112876). Funding bodies did not have a role in the design of the study and collection, analysis, and interpretation of data and in writing the manuscript.

## Authors' Contributions

A.L.C. was involved in conceptualization, formal analysis, methodological development, drafting the manuscript, and funding acquisition. K.J.N. was involved in formal analysis, drafting the manuscript, drafting figures, and validation. S.Z. and M.G.M. were involved in validation, supervision, data curation, and funding acquisition. D.B.F. was involved in validation, supervision, and funding acquisition. All authors read and approved the final manuscript.

## Supplementary Material

giaa091_GIGA-D-20-00045_Original_Submission

giaa091_GIGA-D-20-00045_Revision_1

giaa091_GIGA-D-20-00045_Revision_2

giaa091_GIGA-D-20-00045_Revision_3

giaa091_Response_to_Reviewer_Comments_Original_Submission

giaa091_Response_to_Reviewer_Comments_Revision_1

giaa091_Response_to_Reviewer_Comments_Revision_2

giaa091_Reviewer_1_Report_Original_Submission Aniket Mishra -- 4/8/2020 Reviewed

giaa091_Reviewer_2_Report_Original_Submission Hon Cheong So -- 4/28/2020 Reviewed

giaa091_Reviewer_2_Report_Revision_1 Hon Cheong So -- 6/2/2020 Reviewed

giaa091_Online_Appendix

## Appendix

### Coming up with an *a priori* hypothesis

Prior to applying our method to the PCG dataset discussed in the main text, we applied our method to genetic data obtained from the Prechter Bipolar Cohort, a longitudinal cohort of 1,111 individuals [51]. The University of Michigan's Biomedical Institutional Review Board approved all recruitment, assessment and research procedures (HUM606). Patients provided written informed consent after receiving a complete description of the study. We focused on individuals with bipolar I disorder. Diagnoses of psychiatric illness (e.g., bipolar disorder type I) or lack of psychiatric illness (i.e., control) were determined using the Diagnostic Instrument for Genetic Studies (DIGS), commonly used in psychiatric research [71]. Diagnoses obtained from the DIGS adhered to DSM-IV diagnostic criteria and were confirmed and re-confirmed annually through a consensus of 3 clinicians, resulting in “best estimate” diagnoses. Participants provided whole-blood samples at study intake for genetic testing of specific single-nucleotide polymorphisms (SNPs). Methods pertaining to genetic testing are described in detail elsewhere [72]. Approximately 0.5 million SNPs were analyzed initially, which were then used to impute alleles for other SNPs, resulting in >9.8 million SNPs in total.

For the application of our method, we used the same set of genes and the same gene weights obtained from simulation of the Ashhad and Narayanan model [40]. Gene ranks were obtained starting with 428 individuals with bipolar disorder I and 193 controls without a psychiatric diagnosis. Genetic variation was first analyzed using PLINK software [73] to account for population stratification and outliers. We performed principal component analysis on SNP data and visualized the participant loadings associated with the first 2 principal components. We removed any individuals who could be separated from the main cluster in this 2D space either visually or with *k*-means clustering. This analysis was repeated until there were no participants who could be separated, leaving a total of 377 participants with bipolar disorder I and 167 controls. Gene-level association to bipolar disorder I was measured using MAGMA software [26]. The 10 leading principal components obtained from the final principal component analysis were included as covariates. Gene locations were defined using NCBI Build 38. A total of 18,300 genes were ranked on the basis of the measured association (*P*-value) with bipolar disorder I, with smallest *P*-values ranked closest to 1.

With gene ranks and weights, we performed our weighted gene-set test (GEMB). We again compare our results to an unweighted gene-set test (applying our gene-set test with equal weights) using all 182 genes from the KEGG calcium signaling pathway [55–57]. We also performed a typical over-representation analysis: genes were labeled as significant or not and then a 1-sided Fisher exact test was applied to test for over-representation of significant genes in the KEGG calcium signaling pathway compared to genes not in the KEGG calcium signaling pathway. However, because the significance level of 0.1 adjusted for false discovery rate yielded no significant genes, we labeled the top 1% of genes as significant [59].

Our gene-set test (GEMB) showed moderate support for our hypothesis that intracellular Ca^2+^ concentration is related to bipolar I disorder (*P* = 0.04). By contrast, focusing on the entire KEGG calcium signaling pathway provided little support for the hypothesis that calcium signaling is important to bipolar I (*P* = 0.63 using our method GEMB with equal weights and *P* = 0.24 using a 1-sided Fisher exact test). These results provided the impetus to study intracellular calcium concentrations in the larger PCG dataset.

### References

71. Nurnberger JI, Blehar MC, Kaufmann CA, et al. Diagnostic interview for genetic studies: rationale, unique features, and training. Arch Gen Psychiatry 1994;51(11):849–59.

72. Lee SH, Ripke S, Neale BM, et al. Genetic relationship between five psychiatric disorders estimated from genome-wide SNPs. Nat Genet 2013;45(9):984–94.

73. plink. http://zzz.bwh.harvard.edu/plink/. Accessed 01 Feb 2020.
