# Supplementary material for: Gene-set Enrichment with Mathematical Biology (GEMB)
Source: Gigascience. 2020 Oct 9;9(10):giaa091. doi: 10.1093/gigascience/giaa091 (PMC7546080; doi:10.1093/gigascience/giaa091)
Supplement: giaa091_GIGA-D-20-00045_Revision_3 [file giaa091_giga-d-20-00045_revision_3.pdf]

GigaScience

Gene-Set Enrichment with Mathematical Biology (GEMB)

--Manuscript Draft--

**Manuscript Number:**	GIGA-D-20-00045R3
**Full Title:**	Gene-Set Enrichment with Mathematical Biology (GEMB)
**Article Type:**	Technical Note

Funding Information:

National Institute of Mental Health (K01-MH112876)	Prof. Amy Cochran
Division of Mathematical Sciences (DMS grant 1714094)	Prof. Daniel Forger
Human Frontiers of Science Program (RPG 24/2012)	Prof. Daniel Forger
Richard Tam Foundation (n/a)	Dr. Melvin G McInnis
Heinz C Prechter Bipolar Research Fund (n/a)	Dr. Melvin G McInnis

Abstract:

Background: Gene-set analyses measure the association between a disease of interest and a set of genes related to a biological pathway. These analyses often incorporate gene network properties to account for differential contributions of each gene. We extend this concept further---defining gene contributions based on biophysical properties---by leveraging mathematical models of biology to predict the effects of genetic perturbations on a particular downstream function.

Results: We present a method that combines gene weights from model predictions and gene ranks from genome-wide association studies into a weighted gene-set test. We demonstrate in simulation how such a method can improve statistical power. To this effect, we identify a gene set, weighted by model-predicted contributions to intracellular Ca^{2+} concentration, that is significantly related to bipolar disorder in a small dataset (P =0.04; n=544). We reproduce this finding using publicly-available summary data from the Psychiatric Genetics Consortium (P =1.7×10^{-4} ; n=41,653). By contrast, an approach using a general calcium signaling pathway did not detect a significant association with bipolar disorder (P =0.08). The weighted gene-set approach based on intracellular Ca^{2+} concentration did not detect a significant relationship with schizophrenia (P =0.09; n=65,967) or major depression disorder (P =0.30; n=500,199).

Conclusions: Together, these findings show how incorporating math biology into gene-set analyses might help to identify biological functions that underlie certain polygenic disorders.

**Corresponding Author:**	Amy Cochran<br>University of Wisconsin Madison<br>UNITED STATES
**Corresponding Author Secondary Information:**	
**Corresponding Author's Institution:**	University of Wisconsin Madison
**Corresponding Author's Secondary Institution:**	
**First Author:**	Amy Cochran
**First Author Secondary Information:**	
**Order of Authors:**	Amy Cochran
	Kenneth J Nieser
	Daniel Forger
	Sebastian Zöllner
	Melvin G McInnis

Powered by Editorial Manager® and ProduXion Manager® from Aries Systems Corporation

**Order of Authors Secondary Information:**	
**Response to Reviewers:**	My apologies for missing the RRID and biotools ID. They have been added.
**Additional Information:**	
**Question**	**Response**
Are you submitting this manuscript to a special series or article collection?	No
**Experimental design and statistics**<br><br>Full details of the experimental design and statistical methods used should be given in the Methods section, as detailed in our Minimum Standards Reporting Checklist. Information essential to interpreting the data presented should be made available in the figure legends.<br><br>Have you included all the information requested in your manuscript?	Yes
**Resources**<br><br>A description of all resources used, including antibodies, cell lines, animals and software tools, with enough information to allow them to be uniquely identified, should be included in the Methods section. Authors are strongly encouraged to cite Research Resource Identifiers (RRIDs) for antibodies, model organisms and tools, where possible.<br><br>Have you included the information requested as detailed in our Minimum Standards Reporting Checklist?	Yes
**Availability of data and materials**<br><br>All datasets and code on which the conclusions of the paper rely must be either included in your submission or deposited in publicly available repositories (where available and ethically appropriate), referencing such data using a unique identifier in the references and in	Yes

the “Availability of Data and Materials” section of your manuscript.<br><br>Have you have met the above requirement as detailed in our Minimum Standards Reporting Checklist?	

Manuscript
Click here to access/download;Manuscript;Gene_set__Giga_R2_.pdf

GigaScience, 2017, 1–11

doi: xx.xxxx/xxxx
Manuscript in Preparation
Paper

PAPER

Gene-Set Enrichment with Mathematical Biology (GEMB)

Amy L Cochran[1,2*], Kenneth J Nieser[2], Daniel B Forger[3], Sebastian Zöllner[4,5] and Melvin G McInnis[5]

[1]Department of Math, University of Wisconsin - Madison and [2]Department of Population Health Sciences, University of Wisconsin - Madison and [3]Department of Mathematics and Department of Computational Medicine and Bioinformatics, University of Michigan and [4]Department of Biostatistics, University of Michigan and [5]Department of Psychiatry, University of Michigan

*cochran4@wisc.edu

Abstract

Background: Gene-set analyses measure the association between a disease of interest and a *set* of genes related to a biological pathway. These analyses often incorporate gene network properties to account for differential contributions of each gene. We extend this concept further—defining gene contributions based on biophysical properties—by leveraging mathematical models of biology to predict the effects of genetic perturbations on a particular downstream function.
Results: We present a method that combines gene *weights* from model predictions and gene *ranks* from genome-wide association studies into a weighted gene-set test. We demonstrate in simulation how such a method can improve statistical power. To this effect, we identify a gene set, weighted by model-predicted contributions to intracellular Ca^{2+} concentration, that is significantly related to bipolar disorder in a small dataset (P=0.04; n=544). We reproduce this finding using publicly-available summary data from the Psychiatric Genomics Consortium (P=1.7×10^{-4}; n=41,653). By contrast, an approach using a general calcium signaling pathway did not detect a significant association with bipolar disorder (P=0.08). The weighted gene-set approach based on intracellular Ca^{2+} concentration did not detect a significant relationship with schizophrenia (P=0.09; n=65,967) or major depression disorder (P=0.30; n=500,199).
Conclusions: Together, these findings show how incorporating math biology into gene-set analyses might help to identify biological functions that underlie certain polygenic disorders.

Key words: Mathematical biology; gene ontology; genetic enrichment; gene-set analysis; bipolar disorder; calcium signaling

Background

Genetic contributions to disease can be complex and might involve the coordination of a collection of genetic variants in the disruption of one or many biological pathways. Previous studies of psychiatric conditions provide evidence that a single genetic variant often confers little disease risk despite high heritability [1, 2, 3]. Rather, psychiatric disorders can be *polygenic* [4]—hundreds to thousands of genes of very small effect contribute to the disorder. For this reason, genetic risk for an individual is commonly measured by aggregating information from multiple genes into a *polygenic* risk score [5, 6, 7, 8, 9]. Each of these variants might play a small role in the disruption of a pathway, but collectively lead to the development of disease. Consequently, uncovering genetic influences on psychiatric disorders can be challenging when etiology of disease depends on more than one gene [10]. Computational approaches are emerging to better prioritize candidate genes [11, 12, 13, 14, 15, 16, 17, 18, 19, 20, 21, 22].

Gene-set analyses are a common tool for measuring the association between a disorder and a *set* of genes rather than a single gene [23, 24, 25, 26, 27, 28]. Many statistical tests

Compiled on: August 6, 2020.
Draft manuscript prepared by the author.

and software are available to perform gene-set analysis (cf. [29, 28]) to determine whether genes in a particular gene set are significantly associated with a phenotype (self-contained) or whether a phenotype is more strongly associated with genes in a set than genes not in the set (competitive) [26, 28, 30]. Often gene sets are defined based on genes that contribute to a particular biological pathway, which enables identification of pathways that are important for a disorder. This approach likely leads to stronger, more reproducible findings if abnormal pathways are what ultimately contributes to genetic risk [31, 28].

However, biological functions may ultimately drive risk as opposed to an abnormal pathway or single gene variant. Biological functions do not map one-to-one to biological pathways; a function can recruit *some* genes from *multiple* pathways [32]. In bipolar disorder, for example, spontaneous neuronal firing rate differs in stem cells derived from bipolar individuals compared to controls [33, 34]. This cellular function—neuronal firing rate—recruits genes from calcium-mediated signaling (GO:0019722), regulation of action potential (GO:0098900), and chemical synaptic transmission (GO:0007268), among others. Hence, if perturbed biological functions drive disease risk, jointly testing genes in one pathway that includes genes of little impact and ignoring genes in other relevant pathways would result in a less powerful gene-set analysis.

Moreover, some genes or gene products play a larger role in the realization of the biological function. To account for this, some gene-set analyses incorporate information about the network structure of gene interactions [12, 13, 14, 15, 35, 36, 37]. **Network-based methods, such as EnrichNet[13], GANPA[37], and LEGO[36], represent the network of functional interactions between genes as a network—a mathematical object made up of vertices (genes or proteins) and edges (connections). Based on features of the network, such as the number of connections a gene has with other genes, genes are given more or less weight in the gene-set test statistic. While these methods attempt to account for the functional non-equivalence of genes, they rely on mathematical properties of the network rather than biological mechanisms involved in the related biological function. The connections between genes might vary in strength or operate in a non-linear way. For example, a gene with many weak connections to other genes might carry equal or less downstream biological influence than a gene with few, strong connections to other genes. This discrepancy would be obscured by considering only the number or pattern of connections.** Greater specificity can be achieved quickly through detailed mathematical models from math biology, which are driven from bottom-up biophysical principles. Efforts within the field have culminated in ModelDB [38] which hosts over 1000 publicly-available models [39]. Examples include models of the hypothalamic-pituitary-adrenal axis, monoamine systems, and circadian rhythms, among others. Model parameters related to genes can be varied to measure the relative contribution of genes to a specific biological function of interest (e.g., firing rate). Incorporating model predictions into gene-set tests might strengthen the link between genes and disorders.

We present a simple method (GEMB: Gene-set Enrichment with Math Biology) for measuring the association between a disorder and genes connected to a biological function, based on model predictions. Our method relies on (i) *ranking genes* in decreasing order of association strength to a disorder and (ii) *assigning weights* to a set of genes to reflect their relative contribution to a specific biological function. We illustrate one approach to assigning weights by using pre-existing models from math biology. Ranks and weights are combined into a test for significance of the association between genes related to a biological function (as predicted by a neurobiological model) and a disorder.

To demonstrate the utility of our method, we test the hypothesis that genes affecting intracellular Ca^{2+} concentration are related to bipolar disorder by incorporating a detailed model of intracellular Ca^{2+} concentrations [40]. Bipolar disorder is a severe and chronic psychiatric disorder [41] with estimated heritability at 85% [42]. Genome-wide association studies report several susceptibility loci [43], including a voltage-gated calcium gene [44], which remains among the strongest findings to date. Calcium signaling is an incredibly complex process to model [45] but has been implicated in many human diseases [46], including bipolar disorder.

Method description

A weighted gene-set statistic

We assume a general set-up of a competitive gene-set test: individuals are phenotyped and analyzed for expression in n genes; each gene is measured for association to the phenotype; and a subset of m genes are determined to be of interest (see Fig 1 for an overview). From this set-up, we require only the rank of each gene in decreasing order of association strength to the phenotype; genes that are most strongly associated with the phenotype have the highest rank (i.e, closest to 1) and those that are most weakly associated with the phenotype have the lowest rank (i.e., closest to n).

We diverge from many gene-set tests by requiring that non-negative weights are assigned to individual genes in the subset of interest. Formally, we require:

- genes labeled 1 to n;
- rank $r_i \in \{1, \ldots, n\}$ for each gene $i = 1, \ldots, n$;
- gene set $\mathcal{S} \subseteq \{1, \ldots, n\}$; and
- weights $w_i \geq 0$ for each gene $i \in \mathcal{S}$ with $\sum_{\mathcal{S}} w_i > 0$.

Without any loss of generality, we assume weights w_i sum to one—we can always re-scale weights so that they sum to one. Then, we define the following test statistic using a weighted sum of the ranks r_i ($i \in \mathcal{S}$):

$$v := \sum_{i \in \mathcal{S}} w_i r_i.$$

The choice of weights encodes an *a priori* hypothesis about the relative contribution of a gene to the phenotype. As a specific case, we can recover an unweighted gene-set test by setting $w_i := 1/m$. This choice of weights captures the *a priori* hypothesis that each gene in $\mathcal{S}$ contributes equally to the phenotype (or a lack of support for one gene over another). In this case, the statistic v is the average rank of the genes in $\mathcal{S}$. Recalling that a rank of one is assigned to the gene with the strongest association, a value $v < \frac{n+1}{2}$ reflects that genes in $\mathcal{S}$ are ranked higher *on average* relative to genes not in $\mathcal{S}$. Conversely, a value $v > \frac{n+1}{2}$ reflects that genes in $\mathcal{S}$ are ranked lower *on average* relative to genes not in $\mathcal{S}$. If $v = \frac{n+1}{2}$, genes in $\mathcal{S}$ are neither ranked higher nor lower on average relative to genes not in $\mathcal{S}$. In other words, small v suggests an association between the gene set and phenotype. We point out that genes in $\mathcal{S}$ do not need to be evenly distributed in rank to achieve $v \approx \frac{n+1}{2}$; they could be disproportionately ranked close to the average rank $\frac{n+1}{2}$ or ranked close to the extreme ranks 1 and n.

As another specific case, we could recover a single gene test by setting $w_j = 1$ for some $j \in \mathcal{S}$ and setting all other weights to zero. This choice captures the *a priori* hypothesis that gene i specifically contributes to the phenotype. The statistic v would be the rank of gene i. More broadly, setting any weight to zero

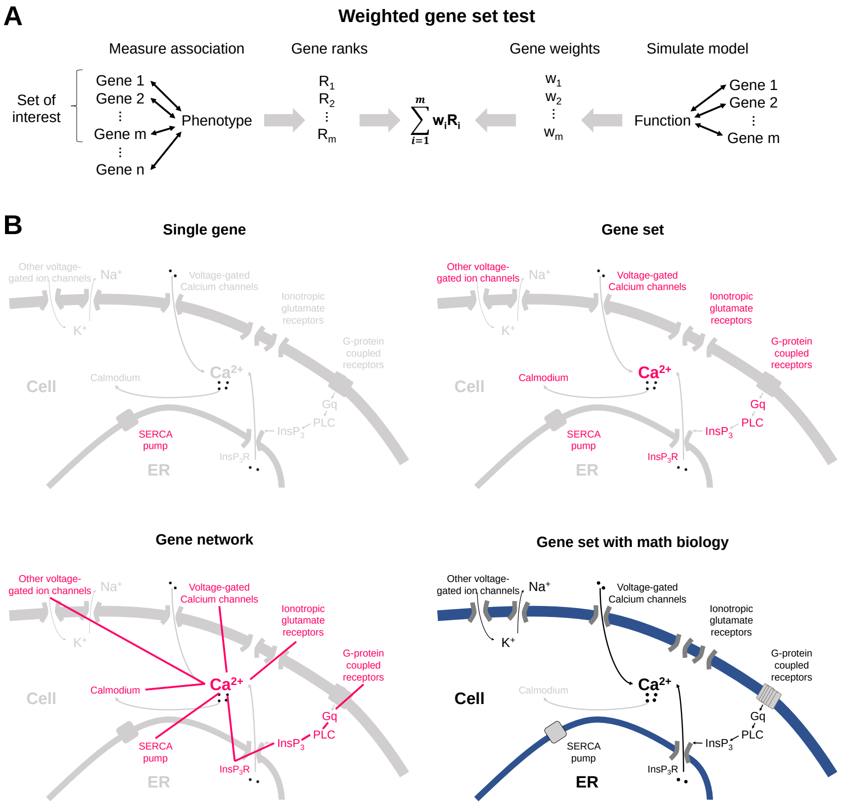

Figure 1. Overview of gene set analysis with math biology. **A)** Genes are ranked based on their association with a phenotype and weighted based on their model-predicted contribution to a specific function. Ranks and genes are combined to perform a weighted gene set test. **B)** Genetic analysis can be performed at the level of either a single gene, a gene set, a gene network, or a gene set connected by math biology. Gene-set analysis with math biology uses models to describe connections between genes based on biophysical principles.

reflects the hypothesis that the corresponding gene does not contribute to the phenotype. The statistic v would be identical in value if we had simply removed the gene from $\mathcal{S}$. Further, smaller weights means a smaller contribution to v.

With more general weights, the statistic v is interpreted similarly to the unweighted version, replacing an average of the ranks with a weighted average. Our interpretation is inherited from the fact that $v - (n+1)/2$ changes sign when genes are ranked in opposite order and increases when a gene in $\mathcal{S}$ is exchanged for a gene not in $\mathcal{S}$ with higher rank. Thus, small v can be thought of as genes in $\mathcal{S}$ have a higher (weighted) relative rank to genes not in $\mathcal{S}$.

A weighted gene-set test

To use v in a statistical test, we must specify a null distribution. For many gene-set analyses, a common null hypothesis is that the genes in $\mathcal{S}$ were chosen uniformly at random from the entire set of genes. Under this null hypothesis, we can construct a null distribution for v by drawing ranks for genes in our set, R_i for $i \in \mathcal{S}$, uniformly at random from

$$\{1, 2, \ldots, n\}$$

without replacement and calculating

$$V := \sum_{i \in \mathcal{S}} w_i R_i.$$

The distribution of the random variable V serves as the null distribution for v.

The alternative hypothesis is that genes in $\mathcal{S}$ were not chosen uniformly at random. In broad terms, they were chosen because of their relationship to the phenotype. Hence, we are interested in how often V with gene ranks chosen randomly suggests a stronger association between set $\mathcal{S}$ and a phenotype than the statistic v determined by the actual association to the phenotype. In other words, we use the probability, or P-value, associated with a one-sided test given by

$$\mathbb{P}(V \le v)$$

to determine whether v is significant. Note, a two-sided test could also be defined by using

$$\mathbb{P}\left(\left|V - \frac{n+1}{2}\right| \ge \left|v - \frac{n+1}{2}\right|\right)$$

A simple way to estimate $\mathbb{P}(V \le v)$ is to use Monte Carlo simulation, where V is repeatedly sampled from its distribution and we count how often a sample of V is less than or equal to v. This computation benefits from the fact that V is simple to calculate and can be sampled in parallel. The law of large numbers ensures a Monte Carlo estimate of $\mathbb{P}(V \le v) = \mathbb{E}(1_{V \le v})$ is unbiased and has variance $\Pr(V \le v)/k$ where k is the number of Monte Carlo samples.

Asymptotic approximation

Alternatively, we could estimate $\mathbb{P}(V \le v)$ with

$$\Phi\left(\frac{v - \mu}{\sigma_W}\right)$$

where Φ is a standard normal distribution and $\mu = \frac{n+1}{2}$ and $\sigma_W^2 = \frac{n^2-1}{12} \sum_{i \in \mathcal{S}} w_i^2$. This approximation follows by making the

Table 1. Difference between Monte Carlo estimates of a one-sided P-value $\mathbb{P}(V \leq v)$ for the weighted gene-set test and estimates using a normal approximation. Weights were defined as $w_i \propto i^l$ $(i = 1, \ldots, m)$ for various l, assuming $\mathcal{S} = \{1, \ldots, m\}$. A total of 10^7 Monte Carlo samples were used in each case.

			v			
l	m	n	$\mu - 4\sigma_w$	$\mu - 3\sigma_w$	$\mu - 2\sigma_w$	$\mu - \sigma_w$
0.5	10	1000	-3.0E-05	-5.7E-04	-1.2E-03	2.2E-03
0.5	10	10000	-2.9E-05	-5.3E-04	-8.0E-04	3.0E-03
0.5	100	1000	-2.1E-05	-5.5E-04	-4.7E-03	-1.1E-02
0.5	100	10000	-8.0E-06	-1.2E-04	-6.1E-04	-7.2E-04
1	10	1000	-3.1E-05	-7.4E-04	-1.5E-03	3.8E-03
1	10	10000	-3.1E-05	-7.0E-04	-1.1E-03	4.4E-03
1	100	1000	-1.8E-05	-5.0E-04	-4.0E-03	-8.7E-03
1	100	10000	-9.2E-06	-9.9E-05	-5.1E-04	-5.6E-04
2	10	1000	-3.2E-05	-1.1E-03	-2.4E-03	6.7E-03
2	10	10000	-3.2E-05	-1.1E-03	-2.1E-03	7.0E-03
2	100	1000	-1.7E-05	-4.4E-04	-3.1E-03	-6.2E-03
2	100	10000	-8.2E-06	-1.6E-04	-4.0E-04	-7.3E-05

simplifying assumption that ranks R_i are drawn uniformly at random from $\{1, \ldots, n\}$ *with* replacement (as opposed to without replacement) and then noting that the resulting V is a sum of independent random variables with respective means $w_i \frac{n+1}{2}$ and variances $w_i^2 \frac{n^2-1}{12}$ $(i \in \mathcal{S})$. Table 1 compares Monte Carlo estimates of one-sided P-values to estimates using a normal approximation.

Type I error and power

Type I error is controlled by the distribution of gene ranks under the null hypothesis of no association between the gene set and the phenotype. Our weighted gene-set test uses the null distribution that arises when any permutation of gene ranks is equally likely. However, the true distribution of gene ranks when there is no association is not clearly defined due to the complex correlations that might exist among genes. Moreover, the null distribution of gene ranks is determined by the method used to measure gene-phenotype associations (see [47] for a comparison). It is thus important to choose a method for ranking genes that properly controls Type I error.

Power can be improved with a weighted gene-set test over gene-set or single gene analyses when multiple genes have differential contribution to disease risk. To illustrate, consider n genes and a set of two independent genes with very small association to the disease. Under our null hypothesis, gene ranks divided by n are approximately uniformly distributed between 0 and 1. A single gene test could assess whether or not each gene's normalized rank is below some critical value (gray region; Fig 2A). By contrast, a gene-set test could assess whether or not the sum of the two genes' normalized ranks is below some threshold (blue region; Fig 2A) and a weighted gene-set test could assess whether or not a weighted sum of the two genes' normalized ranks is below some threshold (green region; Fig 2A). In each case, Type I error is controlled at 0.05 when the rejection region has an area of 0.05.

To estimate statistical power, we consider a situation when two independent genes of interest are ranked based on an F-test examining if ν coefficients are zero when regressing phenotype on gene variables, as is done in MAGMA with ν being the number of gene-level principal components used in the regression model [26]. For simplicity, we set $\nu = 10$ and assume that the P-value for each gene recovered from the F-test would be its rank normalized by the number of genes n. For a sample size of k, the test statistic for gene 1 and 2 would follow an F-distribution with $\nu - 1$ and $k - \nu$ degrees of freedom under the null hypothesis (no gene-phenotype association). For an alternative distribution, we assume that the test statistic follows a *non-central* F-distribution with $\nu - 1$ and $k - \nu$ degrees of freedom and non-centrality parameters kd_1 or kd_2 for gene 1 and 2, respectively. Under this alternative, increasing sample size or non-centrality leads to larger joint densities for normalized ranks near the axes (Fig 2B). Thus with only 2 genes, these changes can improve statistical power—the probability of arriving at normalized ranks that lie in each reject region (Fig 2C). We expect that this improvement would continue to hold or grow with increases in gene set size and increasingly differential effect sizes. Hence, this example provides support that weighting normalized gene ranks can further increase statistical power by accounting for differential contributions of genes to a disease.

Gene correlation

As alluded to above, nearby genes are often correlated. This correlation could lead to correlated gene ranks and subsequently violate the null distribution arising from drawing gene ranks uniformly at random without replacement. To account for gene correlation, we can draw ranks distributed as a multivariate normal random variable $(\epsilon_1, \ldots, \epsilon_n)$ with mean zero and covariance matrix Σ and let R_i be the rank of ϵ_i among the set $\{\epsilon_1, \ldots, \epsilon_n\}$. The covariance matrix Σ captures gene correlation. The resulting distribution for $V = \sum_{i \in \mathcal{S}} w_i R_i$ can then be estimated by Monte Carlo simulation as before.

Determining gene weights

Weights can capture any *a priori* hypothesis whether justified by functional data, literature surveys, or experiments. Our goal, however, is more specific: we want weights to reflect the relative contribution of genes to a specific biological function. If we have reason to think certain genes play a large role in the biological function of interest, we upweight them. If genes do not affect the biological function of interest, we downweight them. In this way, our weighted gene-set test incorporates the hypothesis that a specific biological function (captured by the weights) is important to a phenotype.

To inform the choice of weights, we propose a general approach using models from math biology. We start with a neurobiological model that can return a scalar measure of the function of interest. As noted earlier, many models are publicly available through sources such as ModelDB [38]. Next, we consult gene databases to identify genes related to one or more model parameters and create a mapping of genes to model parameters. Then, we perform a global sensitivity analysis to measure the relative contribution of each parameter to a specific function of interest. We opt for a global sensitivity analy-

A

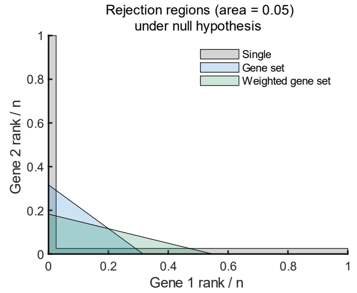

B

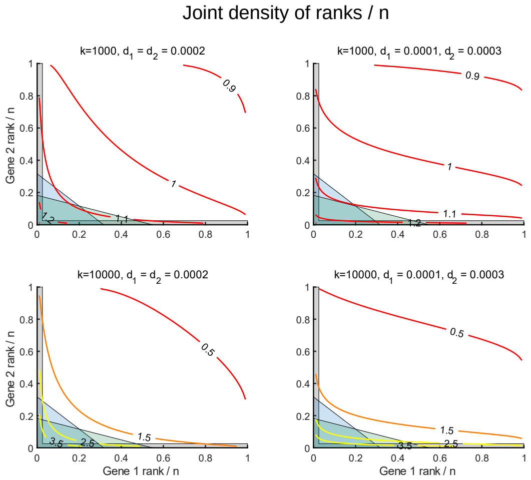

C

Sample size k	Non-centrality factor		Power		
	Gene 1 d_1	Gene 2 d_2	Single[a]	Gene set (w_1=w_2=0.5)	Weighted gene set ($3w_1$=w_2=0.75)
1000	0.0002	0.0002	0.0572	**0.0576**	0.0576
1000	0.0001	0.0003	0.0573	0.0576	**0.0586**
10000	0.0002	0.0002	0.143	**0.153**	0.151
10000	0.0001	0.0003	0.150	0.147	**0.164**

[a] Corrected for multiple testing

Figure 2. Statistical power. **A)** Possible regions to reject null hypothesis for a single gene test (corrected for multiple testing), a gene set test, and a weighted gene set test. **B)** Joint density functions of ranks divided by n for different sample sizes (n) when genes have very small effect sizes (d_1 and d_2). **C)** Statistical power estimated for each case in **(B)**.

sis based on the partial rank correlation coefficient (PRCC) [48] due to its simplicity. Last, we assign weights to each gene based on the contributions of the model parameter to which it is mapped.

We remark that the association between genes and parameters need not be one-to-one. On one hand, models might not be sufficiently detailed to capture the individual contribution of each gene, so multiple genes may be associated with a single parameter. For example, four genes are known to modulate formation of L-type Ca^{2+} channels, but most mathematical models with L-type ion channels do not include individual parameters to capture the differential contributions of each gene. On the other hand, multiple parameters might be associated with a single gene. For example, models of neuronal action potential often distinguish between sodium currents and persistent sodium currents [49] even though both currents may be regulated by the same gene [50]. We describe how we handled these issues in the context of our case study.

Analyses

To illustrate our method, we explore the hypothesis that genes contributing to intracellular Ca^{2+} concentrations in excitable neurons are related to bipolar disorder. Calcium signaling has been both implicated in bipolar disorder and extensively modeled. Furthermore, this hypothesis was initially tested using our method with a relatively small dataset (n=544) from the Prechter Bipolar Cohort [51] (details in Appendix). Thus, the results reported here reproduce our initial finding and validate an *a priori* hypothesis with a much larger dataset.

Gene ranks

Summary genetic data were obtained on subjects with bipolar disorder (n=20,129) and controls (n=21,524) from the Psychiatric Genomics Consortium (PGC) [52, 53]. Association was measured between 8,958,989 SNPs and bipolar disorder, resulting in P-values for each SNP. Data collection and analysis are detailed in Ruderfer et al [53]. Using SNP-level summary data, gene-level association with bipolar disorder was measured using MAGMA software [26]. Default parameter settings were used in MAGMA, with gene boundaries defined based on NCBI Build 37 (hg19). A total of 3,554,879 (39.68%) SNPs mapped to at least one gene, whereas 18,309 genes (out of 19,427 genes) mapped to at least one SNP. Linkage disequilibrium between SNPs was estimated by MAGMA using reference data files created from Phase 3 of 1,000 Genomes [54]. The set of 18,309 genes were ranked based on their measured association (P-value) with bipolar disorder; the smallest P-values were ranked closest to 1.

Genes weights

We used a detailed model of an intracellular Ca^{2+} concentration in a hippocampus CA1 pyramidal cell developed by Ashhad and Narayanan in [40]. The model is publicly-available in ModelDB (Model 150551) and written with free Neuron software [38]. Furthermore, it captures key contributors to intracellular Ca^{2+} concentrations, including ion transport (K^+, Na^+, and Ca^{2+}) across the cell membrane; transport of Ca^{2+} into and out of the sarcoplasmic endoplasmic reticulum; synaptic plasticity; and mediating receptors such as inositol triphosphate ($InsP_3$), ionotropic glutamate receptors, and metabotropic glutamate recptors (mGR). Finally, the model uses a morphologically realistic three-dimensional representation of a hippocampus CA1 pyramidal cell accompanied by spatial dynamics giving rise to Ca^{2+} waves.

To identify genes of interest, we started with 182 genes making up the *Calcium signaling pathway* (Pathway ko04020) in the Kyoto Encyclopedia of Genes and Genomes (KEGG) [55, 56, 57]. Each gene was evaluated for whether it could modulate intracellular Ca^{2+} concentrations in the model using the Gene database from the National Center for Biotechnology Information [58]. A total of 38 genes could modulate intracellular Ca^{2+} concentrations in the model, by way of ion channels, ion pumps, or receptors. We found three ion channels (Na^+, A-type K^+, and delayed rectifying K^+) and two receptors (NMDA and AMPA) that could affect intracellular Ca^{2+} concentration in the model but had not been associated with genes in the KEGG Calcium signaling pathway. An additional 31 genes were found related to these channels or receptors. Of the 69 genes identified, 4 genes (ATP2B3,CACNA1F,GRIA3,KCND1) were excluded, because they were not associated with gene ranks (described below). A total of 65 genes were analyzed.

For each gene, we identified a parameter that could modulate (up and down) the modeling component related to the gene. For example, channel conductance was associated with

Table 2. Calcium genes and associated model parameters. Calcium genes impact either ion channels, ion pumps, or receptors in the Ashhad and Narayanan model [40]. Baseline parameter values were taken from [40].

Genes	Value	Parameter
ATP2B[1–2,4]	0.008 μM ms^{-1}	Average rate γ_0 of Ca^{2+} flux density
ATP2A[1–3]	0.1 μM ms^{-1}	Amplitude V_{max} of SERCA pump uptake
CACNA1[C–D,S]	0.316 mS cm^{-1}	L-Type Ca^{2+} channel conductance g_{CaL}
CACNA1[G–I]	0.1 mS cm^{-1}	T-Type Ca^{2+} channel conductance g_{CaT}
GRM[1,5]	0.3e-3	Metabotropic glutamate receptor density $[mGR]_0$
GNA[Q,11,14–15]	100 ms^{-1}	G_α-bound activated PLC formation rate k_7
PLC[B1–B4,D1,D3–D4,E1,G1–G2,Z1]	0.83 ms^{-1}	PLC_α-bound PIP_2 formation rate k_9
ITPR[1–3]	1.85	IP3 receptor density $\bar{g}_{InsP_3R}$
GRIN[1,2A–2D,3A–3B]	1.938107025 nM s^{-1}	Maximum NMDA receptor permeability $\bar{P}_{NMDA}$
GRIA[1–2,4]	1.29207135 nM s^{-1}	Maximum AMPA receptor permeability $\bar{P}_{AMPA}$
KCN[A4,C3–C4,D2–D3]	22 mS cm^{-2}	A-type K^+ channel conductance g_{KA}
KCN[A1–A3,A6–A7,B1–B2,C1–C2]	3 mS cm^{-2}	Delayed rectifying K^+ channel conductance $\bar{g}_{KDR}$
SCN[1–5,8–11]A	90 mS cm^{-2}	Na^+ channel conductance $\bar{g}_{Na}$

ion channel genes. Default parameter values were taken from the simulation in Figure 6 of [40]. Other genes, associated parameters, and default values are summarized in Table 2.

With parameters and genes identified, we used the Ashhad and Narayanan model to simulate intracellular Ca^{2+} concentrations during an established protocol for inducing synaptic plasticity at a synapse, namely 900 pulse stimulation at 10 Hz; see Figure 6 in [40]. We simulated 320 samples of parameter sets using Latin-hypercube sampling from a normal distribution with mean given by the respective baseline parameter in [40], standard deviation given by 5% of the respective baseline parameter, and zero correlation. For each parameter set, we simulated intracellular Ca^{2+} and measured average intracellular Ca^{2+} concentrations during initial transients induced in the first three seconds of the simulation.

We estimated the PRCC between each parameter and the measured concentrations controlling for the remaining parameters (Fig 3). We found, for example, a strong positive partial correlation between average intracellular Ca^{2+} concentrations and maximum permeability $\bar{P}_{NMDA}$ of NMDA receptors and a strong negative partial correlation between average intracellular Ca^{2+} concentrations and the amplitude V_{max} of SERCA pump uptake.

Based on estimated PRCCs, we defined weights for the 65 genes as follows. For each of the N_k genes assigned to parameter k with PRCC ρ_k, we assigned weights $|\rho_k|/N_k$. We then re-normalized weights to sum to one. Note that we could use any function of ρ_k to assign weights to associated genes. We use only the magnitude of PRCC, since measured associations between genes and phenotypes are not sufficiently specific to reflect the direction of association in addition to the magnitude. We divide by the number of genes assigned to parameter k, so that a single component in the model is not weighted heavily simply because there are a large number of genes assigned to the component.

Weighted gene-set test

Combining gene ranks obtained from the genetic analysis with gene weights obtained from the model of calcium signaling, we performed the weighted gene-set test. For comparison, we performed an unweighted gene-set test using all 182 genes from the KEGG Calcium signaling pathway [55, 56, 57] by assigning equal weights to all 182 genes. In addition, we performed a typical over-representation analysis with the set of 182 genes. Genes were labeled as significant or not based on a significance level of 0.1 adjusted for false discovery rate [59] (a significance level 0.0044 for our problem); a one-sided Fisher's exact test was performed to test for over-representation of significant

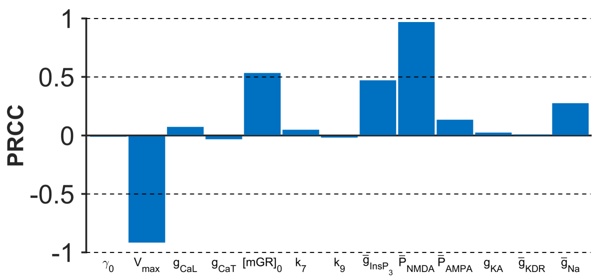

Figure 3. Partial rank correlation coefficient (PRCC) estimated for 13 parameters that modulate intracellular Ca^{2+} concentrations in the Ashhad and Narayanan model [40]. PRCC measures partial correlation between a parameter and the functional measure of interest (average intracellular Ca^{2+} concentration) controlling for the contribution of other parameters.

genes in the KEGG calcium signaling pathway compared to genes not in the KEGG calcium signaling pathway.

Our gene-set test (GEMB) showed strong support for our hypothesis that genes contributing to intracellular Ca^{2+} concentration are related to bipolar disorder (P=1.7$\times$10^{-4}; Fig 4). Further, focusing on the entire KEGG Calcium signaling pathway without consideration of differential contributions to biological function provided little support for the hypothesis that calcium signaling is important to bipolar disorder (P=0.26 using our method GEMB with equal weights and P=0.081 using a one-sided Fisher's exact test). These discrepancies show that incorporating weights could possibly be illuminating biological factors that contribute to a psychiatric disorder.

Sensitivity analyses

Additional analyses were performed to evaluate sensitivity of our result to four factors. First, we wanted to ensure that our test would not simply find intracellular Ca^{2+} concentration to be important for any disease, since such lack of specificity would limit the practical value of our method. An identical procedure used for the bipolar disorder dataset was applied to datasets for schizophrenia (33,426 cases and 32,541 controls) [53] and major depressive disorder (170,756 cases and 329,443 controls) [60] using summary data from the Psychiatric Genomics Consortium (Fig 4). Even though these two disorders have shared genetic risk with bipolar disorder, our weighted gene-set test did not find evidence to support the hypothesis that genes contributed to intracellular Ca^{2+} concentration are related to schizophrenia (P = 0.09) or major depressive disorder (P = 0.30). Neither our method with equal weights

Gene	Functional target	P
ATP2A1	Sarcoplasmic/endoplasmic reticulum calcium ATPase (SERCA)	1.2×10^{-3}
ITPR3	Inositol 1,4,5-trisphosphate (IP_3) receptor	1.1×10^{-3}
ATP2A2	Sarcoplasmic/endoplasmic reticulum calcium ATPase (SERCA)	8.1×10^{-4}
GRIN2A	Ionotropic glutamate receptor [NMDA]	6.6×10^{-4}
GRM1	Metabotropic glutamate receptor	4.9×10^{-4}
GRIN2B	Ionotropic glutamate receptor [NMDA]	4.4×10^{-4}
GRM5	Metabotropic glutamate receptor	4.2×10^{-4}
GRIN3B	Ionotropic glutamate receptor [NMDA]	2.7×10^{-4}
GRIA4	Ionotropic glutamate receptor [AMPA]	2.4×10^{-4}
SCN2A	Voltage-gated sodium channel	2.1×10^{-4}
Baseline		1.7×10^{-4}

Table 3. Top 10 genes contributing to statistical significance of intracellular Ca^{2+} concentrations. Genes are ranked in order of largest P value after applying our gene-set test with the gene removed.

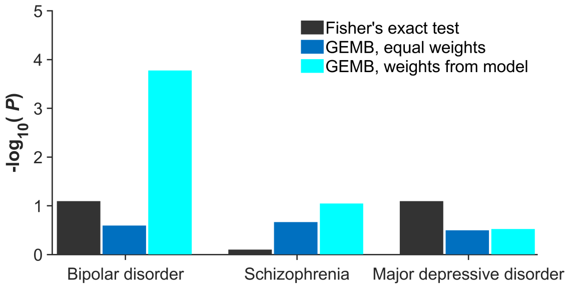

Figure 4. One-sided P-values estimated for gene-set tests. Three tests were performed for each disorder: (1) over-representation test (Fisher's exact test) applied to the KEGG Calcium signaling pathway; (2) our gene-set test (GEMB) with equal weights applied to the entire KEGG Calcium signaling pathway; and (3) our gene-set test (GEMB) with genes related to the Ashhad and Narayanan model [40] and weighted according to their relative contribution to our functional measure of interest (average intracellular Ca^{2+} concentration in an excitable cell).

or a one-sided Fisher's exact test suggested that the entire KEGG Calcium signaling pathway was significantly related to schizophrenia or major depressive disorder (P >0.05). Thus, our weighted gene-set test supports that these findings are specific to bipolar disorder.

Second, we wanted to ensure our result was not driven by a single gene. We performed the weighted gene-set test repeatedly, removing each gene one at a time and recovering a P-value for each test. Regardless of which gene was removed, our test still found that the weighted gene set related to intracellular Ca^{2+} concentration was significantly associated with bipolar disorder. Through this process, we identified a list of 10 genes that contributed the most evidence—based on the largest increases in the P-value recovered after removing the given gene—to the association between the weighted gene set and bipolar disorder (Table 3). The top 10 genes are involved in SERCA pumps, IP_3 receptors, and ionotropic and metabotropic glutamate receptors. We also verified that our result was not driven by the CACNA1C gene, which is important since motivation for studying calcium signaling was driven in part by prior PGC results that implicate CACNA1C. The CANA1C gene alone was significant, ranking 10th out of 18,195 genes (P =10/18195=5.5×10^{-4}), but our gene-set test continued to provide strong support for the remainder of the gene set contributing to intracellular Ca^{2+} with the CACNA1C gene removed (P =1.9×10^{-4}).

Third, we checked sensitivity of our result to gene boundaries as defined by NCBI Build 38, since SNPs outside the gene boundary may still be relevant to the gene. We generated new gene ranks using MAGMA but extended the gene boundary by 10 kilobases in either direction. With these gene ranks, our test still found the weighted gene-set contributing to intracellular Ca^{2+} concentration to be significantly related to bipolar disorder ($P = 9.6 \times 10^{-4}$), albeit to a lesser extent.

Lastly, we checked sensitivity of our result to correlation between genes. An estimated gene correlation matrix was recovered from MAGMA software. Because the resulting matrix was not positive definite, we adjusted the smallest eigenvalues to be at least a value of 10^{-6} leaving the eigenvectors alone. The adjusted gene matrix Σ was then incorporated into the weighted-gene set test to account for gene correlation as described above. When accounting for gene correlation, our weighted gene-set test still found that intracellular Ca2+ concentration was related to bipolar disorder ($P = 1.7 \times 10^{-4}$).

Discussion

We presented a method for examining associations between biological functions and psychiatric disorders which we call GEMB (Gene-set Enrichment with Math Biology). Central to our method are gene weights that measure the relative contribution of a gene to a particular biological function, which we determine using a neurobiological model. We applied our approach to assess the hypothesis that genes involved in the regulation of intracellular Ca^{2+} concentrations are related to bipolar disorder. Gene weights were based on their relative contribution to intracellular Ca^{2+} concentrations, determined by a detailed model of calcium signaling from Ashhad and Narayanan [40]. Gene ranks were obtained using summary genetic data from the Psychiatric Genomics Consortium on bipolar disorder [53], consisting of 20,129 individuals with bipolar disorder and 21,524 controls. Combining gene ranks and weights with our weighted gene-set test, we found strong support for the hypothesis that the gene set contributing to intracellular Ca^{2+} concentrations is related to bipolar disorder (P=1.7×10^{-4}) compared to little support based on a test using the more general KEGG calcium signaling pathway (P=0.081). This result illustrates how gene sets defined based on biological pathways may be too broad to capture the genetic effect on a biological *function* that is associated with a disorder.

A practical benefit of our weighted gene-set test is that only gene ranks are needed from genetic data. Gene ranks can be shared across researchers more easily and require fewer regulatory and computational resources to analyze compared to full genetic data. Sharing genetic resources and data has become the norm in genetic research, as the community moves towards large consortia to achieve the sample sizes, level of evidence, and study consistency that are expected. The Psychiatric Genomics Consortium, for example, has ~300 investigators and >75,000 subjects [61], and the National Institute

of Mental Health (US) has made genetic data available to researchers. Gene ranks can even be recovered from summary data rather than full genetic data as done by MAGMA [26].

Popular gene-set tests typically start with gene-level measures of association between genes and the phenotype like fold change, *t* statistic, *p* value, gene ranks, likelihood ratio, regression coefficient, and correlation coefficient [62, 25, 30, 63]. We transformed *p* values obtained from MAGMA into gene ranks. The choice of gene-level measure and any subsequent transformation is an important decision, since it can alter the findings from gene set analyses in several ways. First, the gene-level measure may yield better accuracy in finding significant gene sets depending on the genetic architecture of the disease of interest [62, 64]. A binary variable may be better than a gene rank for diseases with a few highly influential genes but worse for diseases with a large number of moderately influential genes [64]. Second, gene-level measures differ in their statistical properties. For example, larger sample sizes may change *p* values without affecting gene ranks, and using a probit transformation of *p* values rather than *p* values directly, as in MAGMA, can yield a distribution closer to normal [26]. Third, the choice of statistic could change the clinical relevance of the findings, as one finds in a single gene analysis when comparing fold change to a *t* statistic [65]. Although we use gene ranks, ranks can be easily replaced by any gene-level measure of association if desired. It will be important for future studies to explore how different gene-level measures fare with the weighted gene-set test we propose here.

Once gene ranks are determined, our method then needs only gene weights from neurobiological models, which too has its benefits. Neurobiological models are numerous, experimentally validated, and publicly available in ModelDB [38]. For example, we were able to quickly explore calcium signaling in bipolar disorder, due to the accessibility of a detailed model developed by Ashhad and Narayanan [40] (Model 150551). Similar quick explorations could be used to examine other potentially important biological functions. In searching key words in ModelDB, we found 171 models that contain the concept of *Synaptic Plasticity*, 168 models that contain the concept of *Calcium dynamics*, 47 models that contain the dopamine neurotransmitter, and 9 models that contain the concept of *Circadian Rhythms*, to name a few [38]. Together, these models could annotate genes based on model-predicted functional measures to add to current resources that annotate genes based on biological pathways, such as KEGG [55, 56, 57].

With GEMB, neurobiological models may inform genetic studies, but the reverse may also be true: genetic studies may inform neurobiological models. In psychiatry, for instance, there is growing emphasis on team science, affording many opportunities for researchers from the mathematical sciences to help tackle problems [66]. However, just as it is difficult to pin down genes to study in psychiatric disorders, it is also difficult to pin down specific biological processes to study, since abnormal function is found for many neural systems in a psychiatric disorder [67]. Thus, GEMB could help identify, or ground, candidate neurobiological models for studying in psychiatry. The model of Ashhad and Narayanan [40] provides one such example.

There are a number of other methods like GEMB that try to incorporate information about gene function into gene-set analyses. Network-based methods represent genes as vertices and interactions between two genes as edges resulting in a a mathematical object known as a network [12, 13, 14, 15, 16, 17, 18, 35, 36, 37]. Properties of this network (e.g., their location in the network, number of connections to a gene) are used to adjust the weight given to a gene in a gene-set analysis. In a broad sense, genes might be important to a biological pathway if they are more connected to other genes. For example, GANPA [37] and LEGO [36] builds a network based on a gene co-expression, protein-protein interactions, and gene ontology. Based on this network, they weight each gene in the set of interest as a function of the number of connections to other genes in the set and other genes in the entire network. EnrichNet uses a random walk on these networks to measure associations between genes and target cellular processes. Alternative ways to incorporate functional information about genes include Bayesian approaches that account for overlap between gene sets [19, 20] or approaches based on gene expression levels [21, 22]. A benefit of our method is that it is sufficiently general, such that weights could also be determined from network analysis, experiments or meta-analysis. Weights need only be non-negative and sum to one.

The presented method GEMB was designed to be simple, which has certain limitations. First and foremost, our method like many genetic analyses ignores the various ways that gene and SNP interactions can influence a disease, resulting in a complex genetic architecture of the disease. For example, one gene or part of a gene can regulate another gene, or two genetic variants may lead to increased risk in a disorder that surpasses the additive risk of each variant alone. Hence, our method like other gene-set analyses aims to identify genes that are associated with disorders for further scientific investigation rather than to establish causal relationships between genes and disorders. Second, we do not account for gene interactions in the neurobiological model. Sobol's method of global sensitivity analysis [68], for example, could measure relative contribution of parameters and their higher-order interactions. Our weighted gene-set test could be extended to incorporate these interactions. Third, neurobiological models are sure to be imperfect, meaning that gene weights are only *predicted* measures of biological function. This issue is, of course, common to all modeling. The question then is not whether using a model leads to the correct answer, but rather whether using models to favor certain genes would strengthen inferences compared to treating the genes equally. This is an empirical question that only continued analyses and applications can answer.

In summary, we propose an approach to gene-set analysis that incorporates math biology. Our method can be used flexibly, requiring only that genes are ranked and weighted. Genes can be ranked using any algorithm, even when only summary genetic data are available. Ranks can be determined for any disease. Genes can be weighted using any information whether from experiments, prior analyses, simulation, or math biology—though we focused on the latter. Weights can even be reused from one disease to the next. When the underlying model of math biology is complicated, a researcher could use their own knowledge or borrow weights from another study (e.g., weights for intracellular Ca^{2+} concentration from the present study). These features together with increasing availability of genetic datasets and models leave few barriers to our method's use. In turn, our method may help to improve statistical power in gene-set analyses. Most importantly, it could facilitate meaningful biological interpretations which are ultimately necessary in our understanding of the genetic basis of disease.

Availability of source code and requirements

Lists the following:

- Project name: GEMB
- Project home page: https://github.com/cochran4/GEMB
- RRID: SCR_018904
- biotoolsID: gemb

- Operating system(s): Platform independent
- Programming language: Matlab
- Other requirements: None
- License: GNU GPL

Availability of supporting data and materials

The data sets supporting the results of this article are based on published work on schizophrenia and bipolar disorder as part of the Psychiatric Genomics Consortium; data are available through the Psychiatric Genomics Consortium website [69].

An archival copy of the source code and supporting data is available via the GigaScience database GigaDB [70].

Declarations

List of abbreviations

GEMB: Gene-set enrichment with math biology; KEGG: Kyoto Encyclopedia of Genes and Genomes; PRCC: partial rank correlation coefficient; SNP: single nucleotide polymorphism.

Ethical Approval

For results reported in the Appendix, The University of Michigan's Biomedical Institutional Review Board approved all recruitment, assessment and research procedures (HUM606). Patients provided written informed consent after receiving a complete description of the study.

Consent for publication

Not applicable

Competing Interests

MGM has consulted with and/or received grant funding from Janssen Pharmaceuticals and Takeda Pharmaceuticals; he is a co-owner in Priori-AI, LLC. DBF is the CSO of Arcascope and has equity in the company. Arcascope did not sponsor this research. All other author(s) declare that they have no competing interests.

Funding

This research is supported by the Heinz C Prechter Bipolar Research Fund, the Richard Tam Foundation, a Human Frontiers of Science Program Grant (RPG 24/2012), the National Science Foundation (US; DMS grant 1714094), and the National Institute of Mental Health (US; K01-MH112876). Funding bodies did not have a role in the design of the study and collection, analysis, and interpretation of data and in writing the manuscript.

Author's Contributions

ALC was involved in conceptualization, formal analysis, methodological development, drafting the manuscript, and funding acquisition. KJN was involved in formal analysis, drafting the manuscript, drafting figures, and validation. SZ and MGM were involved in validation, supervision, data curation, and funding acquisition. DBF was involved in validation, supervision, and funding acquisition. All authors read and approved the final manuscript.

References

1. Cardno AG, Gottesman II. Twin studies of schizophrenia: from bow-and-arrow concordances to star wars Mx and functional genomics. American journal of medical genetics 2000;97(1):12–17.
2. Sullivan PF, Kendler KS, Neale MC. Schizophrenia as a complex trait: evidence from a meta-analysis of twin studies. Archives of general psychiatry 2003;60(12):1187–1192.
3. Sullivan PF, Daly MJ, O'donovan M. Genetic architectures of psychiatric disorders: the emerging picture and its implications. Nature Reviews Genetics 2012;13(8):537.
4. Consortium IS, et al. Common polygenic variation contributes to risk of schizophrenia and bipolar disorder. Nature 2009;460(7256):748.
5. Power RA, Steinberg S, Bjornsdottir G, Rietveld CA, Abdellaoui A, Nivard MM, et al. Polygenic risk scores for schizophrenia and bipolar disorder predict creativity. Nature neuroscience 2015;18(7):953.
6. Euesden J, Lewis CM, O'reilly PF. PRSice: polygenic risk score software. Bioinformatics 2014;31(9):1466–1468.
7. Agerbo E, Sullivan PF, Vilhjalmsson BJ, Pedersen CB, Mors O, Børglum AD, et al. Polygenic risk score, parental socioeconomic status, family history of psychiatric disorders, and the risk for schizophrenia: a Danish population-based study and meta-analysis. JAMA psychiatry 2015;72(7):635–641.
8. McIntosh AM, Gow A, Luciano M, Davies G, Liewald DC, Harris SE, et al. Polygenic risk for schizophrenia is associated with cognitive change between childhood and old age. Biological psychiatry 2013;73(10):938–943.
9. Hamshere ML, Langley K, Martin J, Agha SS, Stergiakouli E, Anney RJ, et al. High loading of polygenic risk for ADHD in children with comorbid aggression. American journal of psychiatry 2013;170(8):909–916.
10. Tabor HK, Risch NJ, Myers RM. Candidate-gene approaches for studying complex genetic traits: practical considerations. Nature Reviews Genetics 2002;3(5):391.
11. Zampieri G, Van Tran D, Donini M, Navarin N, Aiolli F, Sperduti A, et al. Scuba: scalable kernel-based gene prioritization. BMC bioinformatics 2018;19(1):23.
12. Draghici S, Khatri P, Tarca AL, Amin K, Done A, Voichita C, et al. A systems biology approach for pathway level analysis. Genome research 2007;17(10):000–000.
13. Glaab E, Baudot A, Krasnogor N, Schneider R, Valencia A. EnrichNet: network-based gene set enrichment analysis. Bioinformatics 2012;28(18):i451–i457.
14. Chen J, Bardes EE, Aronow BJ, Jegga AG. ToppGene Suite for gene list enrichment analysis and candidate gene prioritization. Nucleic Acids Research 2009;37(suppl_2):W305–W311. http://dx.doi.org/10.1093/nar/gkp427.
15. Chen J, Aronow BJ, Jegga AG. Disease candidate gene identification and prioritization using protein interaction networks. BMC bioinformatics 2009;10(1):73.
16. Ala-Korpela M, Kangas AJ, Inouye M. Genome-wide association studies and systems biology: together at last. Trends in genetics 2011;27(12):493–498.
17. Hu T, Sinnott-Armstrong NA, Kiralis JW, Andrew AS, Karagas MR, Moore JH. Characterizing genetic interactions in human disease association studies using statistical epistasis networks. BMC bioinformatics 2011;12(1):364.
18. Fontanillo C, Nogales-Cadenas R, Pascual-Montano A, De Las Rivas J. Functional analysis beyond enrichment: non-redundant reciprocal linkage of genes and biological terms. PloS one 2011;6(9):e24289.
19. Lu Y, Rosenfeld R, Simon I, Nau GJ, Bar-Joseph Z. A probabilistic generative model for GO enrichment analysis. Nucleic Acids Research 2008;36(17):e109. http://dx.doi.org/

10.1093/nar/gkn434.
20. Bauer S, Gagneur J, Robinson PN. GOing Bayesian: model-based gene set analysis of genome-scale data. Nucleic Acids Research 2010;38(11):3523–3532. http://dx.doi.org/10.1093/nar/gkq045.
21. Davies MN, Meaburn EL, Schalkwyk LC. Gene set enrichment; a problem of pathways. Briefings in functional genomics 2010;9(5-6):385–390.
22. Le-Niculescu H, Patel S, Bhat M, Kuczenski R, Faraone S, Tsuang M, et al. Convergent functional genomics of genome-wide association data for bipolar disorder: Comprehensive identification of candidate genes, pathways and mechanisms. American Journal of Medical Genetics Part B: Neuropsychiatric Genetics 2009;150(2):155–181.
23. of the Psychiatric Genomics Consortium CDG, et al. Identification of risk loci with shared effects on five major psychiatric disorders: a genome-wide analysis. The Lancet 2013;381(9875):1371–1379.
24. Holmans P, Green EK, Pahwa JS, Ferreira MA, Purcell SM, Sklar P, et al. Gene ontology analysis of GWA study data sets provides insights into the biology of bipolar disorder. The American Journal of Human Genetics 2009;85(1):13–24.
25. Subramanian A, Tamayo P, Mootha VK, Mukherjee S, Ebert BL, Gillette MA, et al. Gene set enrichment analysis: a knowledge-based approach for interpreting genome-wide expression profiles. Proceedings of the National Academy of Sciences 2005;102(43):15545–15550.
26. de Leeuw CA, Mooij JM, Heskes T, Posthuma D. MAGMA: generalized gene-set analysis of GWAS data. PLoS computational biology 2015;11(4):e1004219.
27. Askland K, Read C, Moore J. Pathways-based analyses of whole-genome association study data in bipolar disorder reveal genes mediating ion channel activity and synaptic neurotransmission. Human genetics 2009;125(1):63.
28. Huang DW, Sherman BT, Lempicki RA. Bioinformatics enrichment tools: paths toward the comprehensive functional analysis of large gene lists. Nucleic acids research 2008;37(1):1–13.
29. Mooney MA, Wilmot B. Gene set analysis: A step-by-step guide. American Journal of Medical Genetics Part B: Neuropsychiatric Genetics 2015;168(7):517–527.
30. Wang L, Jia P, Wolfinger RD, Chen X, Zhao Z. Gene set analysis of genome-wide association studies: methodological issues and perspectives. Genomics 2011;98(1):1–8.
31. Collins AL, Sullivan PF. Genome-wide association studies in psychiatry: what have we learned? The British Journal of Psychiatry 2013;202(1):1–4.
32. Stoney R, Robertson DL, Nenadic G, Schwartz JM. Mapping biological process relationships and disease perturbations within a pathway network. NPJ systems biology and applications 2018;4(1):22.
33. Chen H, DeLong C, Bame M, Rajapakse I, Herron T, McInnis M, et al. Transcripts involved in calcium signaling and telencephalic neuronal fate are altered in induced pluripotent stem cells from bipolar disorder patients. Translational psychiatry 2014;4(3):e375.
34. O'shea KS, McInnis MG. Induced pluripotent stem cell (iPSC) models of bipolar disorder. Neuropsychopharmacology 2015;40(1):248.
35. Chen EY, Tan CM, Kou Y, Duan Q, Wang Z, Meirelles GV, et al. Enrichr: interactive and collaborative HTML5 gene list enrichment analysis tool. BMC bioinformatics 2013;14(1):128.
36. Dong X, Hao Y, Wang X, Tian W. LEGO: a novel method for gene set over-representation analysis by incorporating network-based gene weights. Scientific reports 2016;6:18871.
37. Fang Z, Tian W, Ji H. A network-based gene-weighting approach for pathway analysis. Cell research 2012;22(3):565.
38. ModelDB;. https://senselab.med.yale.edu/modeldb/, accessed DD Month Year.
39. McDougal RA, Morse TM, Carnevale T, Marenco L, Wang R, Migliore M, et al. Twenty years of ModelDB and beyond: building essential modeling tools for the future of neuroscience. Journal of computational neuroscience 2017;42(1):1–10.
40. Ashhad S, Narayanan R. Quantitative interactions between the A-type K+ current and inositol trisphosphate receptors regulate intraneuronal Ca2+ waves and synaptic plasticity. The Journal of physiology 2013;591(7):1645–1669.
41. Belmaker R. Bipolar disorder. New England Journal of Medicine 2004;351(5):476–486.
42. McGuffin P, Rijsdijk F, Andrew M, Sham P, Katz R, Cardno A. The heritability of bipolar affective disorder and the genetic relationship to unipolar depression. Archives of general psychiatry 2003;60(5):497–502.
43. Stahl E, Breen G, Forstner A, McQuillin A, Ripke S, Cichon S, et al. Genomewide association study identifies 30 loci associated with bipolar disorder. biorxiv 2018;p. 173062.
44. Ferreira MA, O'Donovan MC, Meng YA, Jones IR, Ruderfer DM, Jones L, et al. Collaborative genome-wide association analysis supports a role for ANK3 and CACNA1C in bipolar disorder. Nature genetics 2008;40(9):1056.
45. Dupont G, Falcke M, Kirk V, Sneyd J. Models of calcium signalling, vol. 43. Springer; 2016.
46. Hörtenhuber M, Toledo EM, Smedler E, Arenas E, Malmersjö S, Louhivuori L, et al. Mapping genes for calcium signaling and their associated human genetic disorders. Bioinformatics 2017;33(16):2547–2554.
47. de Leeuw CA, Neale BM, Heskes T, Posthuma D. The statistical properties of gene-set analysis. Nature Reviews Genetics 2016;17(6):353.
48. Marino S, Hogue IB, Ray CJ, Kirschner DE. A methodology for performing global uncertainty and sensitivity analysis in systems biology. Journal of theoretical biology 2008;254(1):178–196.
49. Paul JR, DeWoskin D, McMeekin LJ, Cowell RM, Forger DB, Gamble KL. Regulation of persistent sodium currents by glycogen synthase kinase 3 encodes daily rhythms of neuronal excitability. Nature communications 2016;7:13470.
50. Kiss T. Persistent Na-channels: Origin and function: A review János Salánki memory lecture. Acta Biologica Hungarica 2008;59(Supplement 2):1–12.
51. McInnis MG, Assari S, Kamali M, Ryan K, Langenecker SA, Saunders EF, et al. Cohort Profile: the Heinz C. Prechter longitudinal study of bipolar disorder. International journal of epidemiology 2018;47(1):28.
52. Ruderfer DM, Fanous AH, Ripke S, McQuillin A, Amdur RL, Gejman PV, et al. Polygenic dissection of diagnosis and clinical dimensions of bipolar disorder and schizophrenia. Molecular psychiatry 2014;19(9):1017.
53. Ruderfer DM, Ripke S, McQuillin A, Boocock J, Stahl EA, Pavlides JMW, et al. Genomic Dissection of Bipolar Disorder and Schizophrenia, Including 28 Subphenotypes. Cell 2018;173(7):1705–1715.
54. Consortium GP, et al. A global reference for human genetic variation. Nature 2015;526(7571):68.
55. Kanehisa M, Goto S. KEGG: kyoto encyclopedia of genes and genomes. Nucleic acids research 2000;28(1):27–30.
56. Kanehisa M, Sato Y, Kawashima M, Furumichi M, Tanabe M. KEGG as a reference resource for gene and protein annotation. Nucleic acids research 2015;44(D1):D457–D462.
57. Kanehisa M, Furumichi M, Tanabe M, Sato Y, Morishima K. KEGG: new perspectives on genomes, pathways, diseases and drugs. Nucleic acids research 2016;45(D1):D353–D361.

58. Gene;. https://www.ncbi.nlm.nih.gov/gene, accessed DD Month Year.
59. Tarca AL, Bhatti G, Romero R. A comparison of gene set analysis methods in terms of sensitivity, prioritization and specificity. PloS one 2013;8(11):e79217.
60. Howard DM, Adams MJ, Shirali M, Clarke TK, Marioni RE, Davies G, et al. Genome-wide association study of depression phenotypes in UK Biobank identifies variants in excitatory synaptic pathways. Nature communications 2018;9(1):1470.
61. Sullivan PF, Agrawal A, Bulik CM, Andreassen OA, Børglum AD, Breen G, et al. Psychiatric genomics: an update and an agenda. American Journal of Psychiatry 2017;175(1):15–27.
62. Ackermann M, Strimmer K. A general modular framework for gene set enrichment analysis. BMC bioinformatics 2009;10(1):47.
63. O'dushlaine C, Kenny E, Heron EA, Segurado R, Gill M, Morris DW, et al. The SNP ratio test: pathway analysis of genome-wide association datasets. Bioinformatics 2009;25(20):2762–2763.
64. Newton MA, Quintana FA, Den Boon JA, Sengupta S, Ahlquist P, et al. Random-set methods identify distinct aspects of the enrichment signal in gene-set analysis. The Annals of Applied Statistics 2007;1(1):85–106.
65. Witten D, Tibshirani R. A comparison of fold-change and the t-statistic for microarray data analysis. Analysis 2007;1776:58–85.
66. Abbott A. US mental-health chief: psychiatry must get serious about mathematics. Nature News 2016;539(7627):18.
67. Goodwin FK, Jamison KR. Manic-depressive illness: bipolar disorders and recurrent depression, vol. 1. Oxford University Press; 2007.
68. Sobol IM. Global sensitivity indices for nonlinear mathematical models and their Monte Carlo estimates. Mathematics and computers in simulation 2001;55(1-3):271–280.
69. Psychiatric Genomics Consortium;. http://www.med.unc.edu/pgc, accessed DD MONTH YEAR.
70. Cochran A, Nieser K, Forger D, Zöllner S, McInnis M, Supporting data for "Gene-Set Enrichment with Mathematical Biology (GEMB)" GigaScience Database 2020;. http://dx.doi.org/10.5524/100764.
71. Nurnberger JI, Blehar MC, Kaufmann CA, York-Cooler C, Simpson SG, Harkavy-Friedman J, et al. Diagnostic interview for genetic studies: rationale, unique features, and training. Archives of general psychiatry 1994;51(11):849–859.
72. Lee SH, Ripke S, Neale BM, Faraone SV, Purcell SM, Perlis RH, et al. Genetic relationship between five psychiatric disorders estimated from genome-wide SNPs. Nature genetics 2013;45(9):984.
73. plink;. http://zzz.bwh.harvard.edu/plink/, accessed DD Month Year.

Appendix

Coming up with an *a priori* hypothesis

Prior to applying our method to the PCG dataset discussed in the main text, we applied our method to genetic data obtained from the Prechter Bipolar Cohort, a longitudinal cohort of 1,111 individuals [51]. The University of Michigan's Biomedical Institutional Review Board approved all recruitment, assessment and research procedures (HUM606). Patients provided written informed consent after receiving a complete description of the study. We focused on individuals with bipolar I disorder. Diagnoses of psychiatric illness (e.g. bipolar disorder type I) or lack of psychiatric illness (i.e. control) were determined using the Diagnostic Instrument for Genetic Studies, commonly used in psychiatric research [71]. Diagnoses obtained from the DIGS adhered to DSM-IV diagnostic criteria and were confirmed and re-confirmed annually through a consensus of three clinicians resulting in "best estimate" diagnoses. Participants provided whole blood samples at study intake for genetic testing of specific single nucleotide polymorphisms (SNPs). Methods pertaining to genetic testing are described in detail elsewhere [72]. About 0.5 million SNPs were analyzed initially, which were then used to impute alleles for other SNPs resulting in over 9.8 million SNPs in total.

For the application of our method, we used the same set of genes and the same gene weights obtained from simulation of the Ashhad and Narayanan model [40]. Gene ranks were obtained starting with 428 individuals with bipolar disorder I and 193 controls without a psychiatric diagnoses. Genetic variation was first analyzed using PLINK software [73] to account for population stratification and outliers. We performed principal component analysis on SNP data and visualized the participant loadings associated with the first two principal components. We removed any individuals who could be separated from the main cluster in this two-dimensional space either visually or with k-means clustering. This analysis was repeated until there were no participants that could be separated, leaving a total of 377 participants with bipolar disorder I and 167 controls. Gene-level association to bipolar disorder I was measured using MAGMA software [26]. The 10 leading principal components obtained from the final principal component analysis were included as covariates. Genes locations were defined using NCBI Build 38. A total of 18,300 genes were ranked based on the measured association (P-value) with bipolar disorder I, with smallest P-values ranked closest to 1.

With gene ranks and weights, we performed our weighted gene-set test (GEMB). We again compare our results to an unweighted gene-set test (applying our gene-set test with equal weights) using all 182 genes from the KEGG Calcium signaling pathway [55, 56, 57]. We also performed a typical over-representation analysis: genes were labeled as significant or not and then a one-sided Fisher's exact test was applied to test for over-representation of significant genes in the KEGG Calcium signaling pathway compared to genes not in the KEGG Calcium signaling pathway. However, since the significance level of 0.1 adjusted for false discovery rate yielded no significant genes, we labeled the top 1% of genes as significant [59].

Our gene-set test (GEMB) showed moderate support for our hypothesis that intracellular Ca^{2+} concentration is related to bipolar I disorder (P=0.04). By contrast, focusing on the entire KEGG Calcium signaling pathway provided little support for the hypothesis that Calcium signaling is important to bipolar I (P=0.63 using our method GEMB with equal weights and P=0.24 using a one-sided Fisher's exact test). These results provided the impetus to study intracellular calcium concentrations in the larger PCG dataset.

Personal Cover
Click here to access/download;Personal Cover;Giga_CoverLetter_Response.pdf

Population Health Sciences

University of Wisconsin
School of Medicine and Public Health

February 14, 2020

Dear Dr. Hans Zauner,

We thank you and the two reviewers for carefully reviewing our manuscript "Gene-Set Enrichment with Mathematical Biology (GEMB)" (GIGA-D-19-00048) and for the opportunity to improve the paper based on the suggestions. We have completely rewritten the paper, added in additional analyses, and addressed concerns and limitations raised by you and the reviewers. We believe these changes have significantly improved our paper. We hope you agree.

In the space below, we detail how we addressed your major concerns and subsequently how we addressed the concerns of each reviewer.

Thank you for your consideration,

Sincerely,

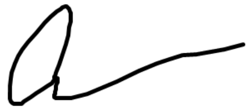

Amy Cochran
Assistant Professor
Population Health Sciences / Math
University of Wisconsin - Madison

Response to Editor

1) As reviewer 2 points out, other recent papers present similar approaches, and in light of these previous publications, the novel contribution of the work is not clear.

We thank you and reviewer 2 for pointing out these papers to us. Indeed, there are a number of genetic analyses that, like our method, account for differential contributions of genes. How we determine these differential contributions is what distinguishes our method. Current methods incorporate the network structure of genes, implicitly assuming that every connection, or edge, between genes is equivalent in nature; however, relationships between genes can differ greatly in strength. A gene with many weak connections to other genes might carry equal or less downstream biological influence than a gene with few, strong connections to other genes. To overcome this limitation, our method uses models from math biology to describe how genes interact based on biophysical principles in an effort to more realistically capture their downstream effects. We accomplish this by

focusing on a particular biological function that is affected by the complex interactions among genes and examining its sensitivity to each gene in the gene set.

In an effort to clarify our novel contribution, we have included a figure (Figure 1) to illustrate these distinctions between our method and other genetic analyses. The abstract has also been updated to include the following:

> "Gene-set analyses measure the association between a disease of interest and a *set* of genes related to a biological pathway. These analyses often incorporate gene network properties to account for differential contributions of each gene. We extend this concept further—defining gene contributions based on biophysical properties—by leveraging mathematical models of biology to predict the effects of genetic perturbations on a particular downstream function."

Our introduction also includes additional details:

> "Moreover, some genes or gene products play a larger role in the realization of the biologic function. To account for this, some gene-set analyses incorporate information about the network structure of gene interactions. However, the nature of the connections between genes might also vary; interactions might operate in a dynamic and nonlinear way. Greater specificity can be achieved quickly through detailed mathematical models from math biology, which are driven from bottom-up biophysical principles."

2) At GigaScience, we aim to highlight methods that can be flexibly adapted to other datasets. In this respect, the reviewers have doubts if and how your approach can be used with other types of data, even by non-mathematicians.

Thank you for pointing out this concern. We added the following concluding paragraph to emphasize the flexibility of our method:

> "In summary, we propose an approach to gene-set analysis that incorporates math biology. Our method can be used flexibly, requiring only that genes are ranked and weighted. Genes can be ranked using any algorithm, even when only summary genetic data are available. Ranks can be determined for any disease. Genes can be weighted using any information whether from experiments, prior analyses, simulation, or math biology — though we focused on the latter. Weights can even be reused from one disease to the next. When the underlying model of math biology is complicated, a researcher could use their own knowledge or borrow weights from another study (e.g., weights for intracellular Ca^{2+} concentration from the present study). These features together with increasing availability of genetic datasets and models leave few barriers to our method's use. In turn, our method may help to improve statistical power in gene-set analyses. Most importantly, it could facilitate meaningful biological interpretations which are ultimately necessary in our understanding of the genetic basis of disease."

We also analyzed two additional datasets (schizophrenia and major depression disorder) to demonstrate our model's flexibility. As alluded to in the above excerpt, we can reuse gene weights from our analysis of calcium signaling and bipolar disorder to analyze the relationship between calcium signaling and other disorders. That is, we do not need to return to the detailed model of intracellular Ca^{2+} concentration when looking at additional datasets if we are interested in the same biological function.

3) Reviewer 1 mentions a couple of technical points that are likely worth exploring further.

We have addressed the technical points raised by Reviewer 1 in our specific response to this review below.

Response to Reviewer #1

1) The manuscript must be proof-read properly.

Department of Population Health Sciences, University of Wisconsin-Madison
610 Walnut Street, 707 WARF, Madison, WI 53726-2397
Phone: 608/263-2880 • Fax: 608/263-2820 • http://www.pophealth.wisc.edu

Our apologies for these mistakes. We have rewritten the manuscript in several places to improve readability and carefully proofread the revised manuscript.

> A) In Abstract, Results section 1st sentence (line 36): In sentence "We present a method to enrich gene-set analyses with models from mathematical biology.", "Enrich gene-set analyses" sounds odd. suggestion "method for gene-set enrichment analyses"

We have removed all references to the odd phrase "enrich gene-set analyses".

> B) There are multiple typos e.g. Method description, A weighted gene-set statistics (line 11-12).

We have corrected the typo you pointed out as well as others.

> C) The Conclusion paragraph is missing in the manuscript.

Thank you for this suggestion. As noted to the editor, we added a concluding paragraph.

> 2) Technical queries
>
> A) Authors use the strength of gene-based association computed using the MAGMA software as a proxy for gene's contribution to disease risk. MAGMA aggregates association statistics of individual common single nucleotide polymorphism (SNP) to compute gene-based p-value. Authors did not discuss that the gene-based association does not mean the involvement of a gene to disease risk. The genetic variant localized within an intron of gene A could be regulating gene B, association of that variant would drive MAGMA derived gene-based p-value of gene A not gene B. Furthermore the genetic risk architecture could be different for various diseases and genes depending on the recombination hotspots/ linkage disequilibrium (LD). Proportion of variants associated with disease could be different for different genes. The gene-based p-values aggregating association test statistics of all variants would show stronger association for genes where large proportion of genetic variants show association with disease than the ones with few variants showing association of disease, irrespective of the variance explained by the region. Authors shall discuss these issues.

Indeed, gene-based association does not guarantee the involvement of a gene to disease risk. This is an important limitation of our method, though we think this limitation is common to most approaches to gene-level association analyses. If better gene-level association analyses become available, they could be used to obtain gene ranks for our method. To address this limitation, we updated the limitations in our manuscript:

> "First and foremost, our method like many genetic analyses ignores the various ways that gene and SNP interactions can influence a disease, resulting in a complex genetic architecture of the disease. For example, one gene or part of a gene can regulate another gene, or two genetic variants may lead to increased risk in a disorder that surpasses the additive risk of each variant alone. Hence, our method like other gene-set analyses aims to identify genes that are associated with disorders for further scientific investigation rather than to establish causal relationships between genes and disorders."

In addition, Reviewer #2 had a related concern about toning down our conclusions. We thus adjusted our conclusions to reflect the point that our method only recovers gene associations and not gene involvement.

As for gene-based *P*-values, MAGMA software, which we used, determines *P*-values by regressing the disease outcome on the principal components of genetic variants of the gene. Thus, it is not necessarily the case that gene association will be overly sensitive to the proportion of genetic variants associated with the disease. For reference, Leoew et al performs a series of analyses in "The Statistical Properties of Gene-Set Analysis" to examine sensitivity of MAGMA and other software to factors such as linkage disequilibrium and gene size.

Lastly, we updated our method in Methods / Weighted Gene Set Test to allow one to adjust for gene-by-gene correlation which can arise from linkage disequilibrium. Based on this new analysis, we added the following analysis in the Results / Sensitivity Analyses section:

> "Lastly, we checked sensitivity of our result to correlation between genes. An estimated gene correlation matrix was recovered from MAGMA software. Because the resulting matrix was not positive definite, we adjusted the smallest eigenvalues to be at least a value of 10^{-6} leaving the eigenvectors alone. The adjusted gene matrix Σ was then incorporated into the weighted-gene set test to account for gene correlation as described above. When accounting for gene correlation, our weighted gene-set test still found that intracellular Ca^{2+} concentration was related to bipolar disorder ($P = 1.7 \times 10^{-4}$)."

B) Authors must describe in detail the parameters they used in deriving gene-based p-values using the MAGMA software notable the gene-boundary.

We used default parameter settings in MAGMA. With default settings, the gene boundary is completely specified by NCBI Build 37 (hg19). We updated the Results / Gene Ranks subsection to include the following sentence:

> "Default parameter settings were used in MAGMA, with gene boundaries defined based on NCBI Build 37 (hg19)."

C) Authors use MAGMA to account for LD between variants within gene, but they did not describe whether they correct for LD between variants in neighboring genes.

In the original paper, we did not correct for LD between variants in neighboring genes. However, since SNPs in nearby genes are correlated, we checked sensitivity to gene boundary definitions to partly account for this correlation. These results have been added to a new section Results / Sensitivity Analysis:

> "Third, we checked sensitivity of our result to gene boundaries as defined by NCBI Build 38, since SNPs outside the gene boundary may still be relevant to the gene. We generated new gene ranks using MAGMA but extended the gene boundary by 10 kilobases in either direction. With these gene ranks, our weighted gene-set test still found intracellular Ca^{2+} concentration to be significantly related to bipolar disorder ($P = 9.6 \times 10^{-4}$), albeit to a lesser extent."

As noted in response to Comment 2A, we also performed an analysis that adjusted for gene-by-gene correlation, which could arise from LD between variants in neighboring genes.

D) Follow-up on point C, in results authors report that the association of calcium signaling with bipolar disorder remain similar even after removing CACNA1C. Did authors also remove neighboring genes CACNA1C? Since the LD block of CACNA1C could still be represented by the neighboring genes.

There is no gene in our gene set that is upstream for CACNA1C and on the same chromosome. The closest gene that is downstream from CACNA1C and on the same chromosome is KCNA6. This gene is over 2 megabases away from CACNA1C, has an estimated correlation of 0.004 with CACNA1C, and was not one of the top 10 genes contributing to the significance of intracellular Ca^{2+} concentrations. Thus, we did not remove genes neighboring CACNA1C from our gene set. However, as noted above, we repeated analyses to account for gene correlation and to widen the gene boundary.

E) Authors shall also discuss the statistical properties of the GEMB method especially control of type-1 error rate.

We updated the manuscript to discuss Type I error rate in the Methods / A Weighted Gene-Set Test subsection:

> "Type I error is controlled by the distribution of gene ranks under the null hypothesis of no association between the gene set and the phenotype. Our weighted gene-set test uses the null distribution that arises when any permutation of gene ranks is equally likely. However, the true distribution of gene ranks when there is no association is not clearly defined due to the complex correlations that might exist among genes. Moreover, the null distribution of gene ranks is determined by the method used to measure gene-phenotype associations (see [46] for a comparison). It is thus important to choose a method for ranking genes that properly controls Type I error."

We also performed a simulation study to evaluate power. A figure (Figure 2) was added to present results from this simulation study. In addition, we added the following two paragraphs in the Methods / A Weighted Gene-Set Test subsection:

> "Power can be improved with a weighted gene-set test over gene-set or single gene analyses when multiple genes have differential contribution to disease risk. To illustrate, consider *n* genes and a set of two independent genes with very small association to the disease. Under our null hypothesis, gene ranks divided by *n* are approximately uniformly distributed between 0 and 1. A single gene test could assess whether or not each gene's normalized rank is below some critical value (gray region; Fig 2A). By contrast, a gene-set test could assess whether or not the sum of the two genes' normalized ranks is below some threshold (blue region; Fig 2A) and a weighted gene-set test could assess whether or not a weighted sum of the two genes' normalized ranks is below some threshold (green region; Fig 2A). In each case, Type I error is controlled at 0.05 when the rejection region has an area of 0.05."

> "To estimate statistical power, we consider a situation when two independent genes of interest are ranked based on an F-test examining if ν coefficients are zero when regressing phenotype on gene variables, as is done in MAGMA with ν being the number of gene-level principal components used in the regression model [18]. For simplicity, we set $\nu = 10$ and assume that the *P*-value for each gene recovered from the F-test would be its rank normalized by the number of genes n. For a sample size of k, the test statistic for gene 1 and 2 would follow an F-distribution with $\nu - 1$ and $k - \nu$ degrees of freedom under the null hypothesis (no gene-phenotype association). For an alternative distribution, we assume that the test statistic follows a *non-central* F-distribution with $\nu - 1$ and $k - \nu$ degrees of freedom and non-centrality parameters kd_1 or kd_2 for gene 1 and 2, respectively. Under this alternative, increasing sample size or non-centrality leads to larger joint densities for normalized ranks near the axes (Fig 2B). Thus with only 2 genes, these changes can improve statistical power—the probability of arriving at normalized ranks that lie in each reject region (Fig 2C). We expect that this improvement would continue to hold or grow with increases in gene set size and increasingly differential effect sizes. Hence, this example provides support that weighting normalized gene ranks can further increase statistical power by accounting for differential contributions of genes to a disease."

F) Authors shall discuss biological aspects of the findings. Apart from CACNA1C are there any other genes that could be prioritized for functional follow-up showing strong association both in terms of genetic risk and molecular relevance?

Thank you for this great suggestion. We added a table (Table 3) showing the top 10 genes identified by our method and added the following paragraph to a new section Results / Sensitivity Analysis:

> "Second, we wanted to ensure our result was not driven by a single gene. We performed the weighted gene-set test repeatedly, removing each gene one at a time and recovering a *P*-value for each test.

Department of Population Health Sciences, University of Wisconsin-Madison
610 Walnut Street, 707 WARF, Madison, WI 53726-2397
Phone: 608/263-2880 • Fax: 608/263-2820 • http://www.pophealth.wisc.edu

Regardless of which gene was removed, our test still found that the weighted gene set related to intracellular Ca^{2+} concentration was significantly associated with bipolar disorder (Table 3). Through this process, we identified a list of 10 genes that contributed the most evidence—based on the largest increases in the *P*-value recovered after removing the given gene—to the association between the weighted gene set and bipolar disorder. The top 10 genes are involved in SERCA pumps, IP_3 receptors, and ionotropic and metabotropic glutamate receptors. We also verified that our result was not driven by the CACNA1C gene, which is important since motivation for studying calcium signaling was driven in part by prior PGC results that implicate CACNA1C. The CANA1C gene alone was significant, ranking 10th out of 18,195 genes ($P = 10/18195 = 5.5 \times 10^{-4}$), but our gene-set test continued to provide strong support for intracellular Ca^{2+} even with the CACNA1C gene removed ($P = 1.9 \times 10^{-4}$)."

Response to Reviewer #2

> 1) With regards to the weighted gene-set analysis (GSA) approach, I wonder whether there are other works addressing a similar problem, given that GSA is a relatively hot topic in bioinformatics/statistical genetics research. For example, from a simple search on my own, a few papers seem to adopt different kinds of gene weightings in their GSA, although the list is not exhaustive. Could the authors discuss and compare their weighted GSA approach with similar/related works in the literature?
>
> References:
> https://www.ncbi.nlm.nih.gov/pmc/articles/PMC3292304/
> https://www.nature.com/articles/srep18871
> http://amp.pharm.mssm.edu/Enrichr/

As detailed in our comments to the editor, we have hopefully addressed this concern.

> 2) The authors considered gene ranks in their approach. However, is it possible to consider the actual (quantitative) GWAS significance when constructing the weighted GSA? Could the authors comment on the pros and cons of either approach?

We could use actual quantitative GWAS significance rather than gene ranks. There was one main reason we opted for gene ranks rather than other possible measures; we commented on this at the end of the second paragraph in the Discussion:

"Other measures of association could even be incorporated into our method if desired. We opted for gene ranks, as opposed to a measure like a *P*-value, in an effort to reduce the sensitivity of our test to sample size."

> 3) The authors applied their method to a Ca signaling pathway and showed its significance in a GWAS on bipolar disorder. The application is interesting, but I am not sure how well or how readily the method may be generalized to tackle, for example, a pathway on synaptic plasticity? For researchers who are not mathematical biologists, how can they make use of data eg in modelDB readily and apply the method described in this paper? If the authors may provide one (or more) examples or how the method can be applied to different types of pathways (other than ion channels), that may help readers to apply their method more readily.

Department of Population Health Sciences, University of Wisconsin-Madison
610 Walnut Street, 707 WARF, Madison, WI 53726-2397
Phone: 608/263-2880 • Fax: 608/263-2820 • http://www.pophealth.wisc.edu

The editor raises a similar concern about the ability of our method to be flexibly used by individuals who are not math biologists. We hope that the concluding paragraph we added has partly addressed your concern. For example, these sentences in this paragraph speak to your concern:

> "…Genes can be weighted using any information whether from experiments, prior analyses, simulation, or math biology — though we focused on the latter. Weights can even be re-used from one disease to the next. So even when the underlying model of math biology could be complicated, a researcher could test the association between a biological function and a disease using their own knowledge or borrowing weights from another study (e.g., weights for intracellular calcium ion concentration from the present study)…"

You are right, however, that models of math biology can be quite complicated and require extensive knowledge of the system of interest. In the long term, our hope is that math biologists would be motivated to propose weights based on their models, since our method provides a way to externally validate the utility of their models. In the meantime, we have determined gene weights for neuronal firing rate based on a model in Paul et al., 2016 ("Regulation of persistent sodium currents by glycogen synthase kinase 3 encodes daily rhythms of neuronal excitability", Nature Communications). These weights are included on our GitHub page. We did not analyze neuronal firing rate in the current paper, since it was not related to a hypothesis we had proposed prior to analyzing the PGC bipolar dataset. We are also in the process of developing weights for extracellular dopamine and serotonin concentrations based on models in Best et al, 2009 ("Homeostatic mechanisms in dopamine synthesis and release: a mathematical model", Theoretical Biology and Medical Modelling) and in Best et al, 2010 ("Serotonin synthesis, release and reuptake in terminals: a mathematical model", Theoretical Biology and Medical Modelling). Synaptic plasticity would be another logical biological function to examine next.

4) As for the abstract, the authors seem to imply that as the p-value for Ca signaling pathway is reduced in the bipolar dataset, there is evidence that the method works in improving pathway inference. Personally I do not fully agree as somehow the evidence of Ca signaling pathways in bipolar disorder and schizophrenia also stems from GWAS studies and the argument is a bit circular. Another reason is that this is a single example (or kind of a "case study") only; while interesting, I think it itself does not serve as strong evidence for the validity of the method as a whole. I would advise the authors to tone down the statement and explain the limitations of their proposed approach in this regard.

Thank you pointing this out. We agree that our results, while interesting, are not sufficient to fully validate our method for reasons that you and the other reviewers have raised. We have thus toned down our statements as you suggested. In addition, we added several analyses (e.g., power study, sensitivity analyses, comparison to schizophrenia and major depressive disorder) to help further validate our method.
