# Supplementary material for: Gene-set Enrichment with Mathematical Biology (GEMB)
Source: Gigascience. 2020 Oct 9;9(10):giaa091. doi: 10.1093/gigascience/giaa091 (PMC7546080; doi:10.1093/gigascience/giaa091)
Supplement: giaa091_Online_Appendix [file giaa091_online_appendix.pdf]

# Appendix

## Coming up with an *a priori* hypothesis

Prior to applying our method to the PCG dataset discussed in the main text, we applied our method to genetic data obtained from the Prechter Bipolar Cohort, a longitudinal cohort of 1,111 individuals [51]. The University of Michigan's Biomedical Institutional Review Board approved all recruitment, assessment and research procedures (HUM606). Patients provided written informed consent after receiving a complete description of the study. We focused on individuals with bipolar I disorder. Diagnoses of psychiatric illness (e.g., bipolar disorder type I) or lack of psychiatric illness (i.e., control) were determined using the Diagnostic Instrument for Genetic Studies (DIGS), commonly used in psychiatric research [71]. Diagnoses obtained from the DIGS adhered to DSM-IV diagnostic criteria and were confirmed and reconfirmed annually through a consensus of 3 clinicians, resulting in "best estimate" diagnoses. Participants provided whole-blood samples at study intake for genetic testing of specific single-nucleotide polymorphisms (SNPs). Methods pertaining to genetic testing are described in detail elsewhere [72]. Approximately 0.5 million SNPs were analyzed initially, which were then used to impute alleles for other SNPs, resulting in $>$9.8 million SNPs in total.

For the application of our method, we used the same set of genes and the same gene weights obtained from simulation of the Ashhad and Narayanan model [40]. Gene ranks were obtained starting with 428 individuals with bipolar disorder I and 193 controls without a psychiatric diagnosis. Genetic variation was first analyzed using PLINK software [73] to account for population stratification and outliers. We performed principal component analysis on SNP data and visualized the participant loadings associated with the first 2 principal components. We removed any individuals who could be separated from the main cluster in this 2D space either visually or with $k$-means clustering. This analysis was repeated until there were no participants who could be separated, leaving a total of 377 participants with bipolar disorder I and 167 controls. Gene-level association to bipolar disorder I was measured using MAGMA software [26]. The 10 leading principal components obtained from the final principal component analysis were included as covariates. Gene locations were defined using NCBI Build 38. A total of 18,300 genes were ranked on the basis of the measured association ($P$-value) with bipolar disorder I, with smallest $P$-values ranked closest to 1.

With gene ranks and weights, we performed our weighted gene-set test (GEMB). We again compare our results to an unweighted gene-set test (applying our gene-set test with equal weights) using all 182 genes from the KEGG calcium signaling pathway [55–57]. We also performed a typical over-representation analysis: genes were labeled as significant or not and then a 1-sided Fisher exact test was applied to test for over-representation of significant genes in the KEGG calcium signaling pathway compared to genes not in the KEGG calcium signaling pathway. However, because the significance level of 0.1 adjusted for false discovery rate yielded no significant genes, we labeled the top 1% of genes as significant [59].

Our gene-set test (GEMB) showed moderate support for our hypothesis that intracellular $Ca^{2+}$ concentration is related to bipolar I disorder ($P = 0.04$). By contrast, focusing on the entire KEGG calcium signaling pathway provided little support for the hypothesis that calcium signaling is important to bipolar I ($P = 0.63$ using our method GEMB with equal weights and $P = 0.24$ using a 1-sided Fisher exact test). These results provided the impetus to study intracellular calcium concentrations in the larger PCG dataset.

## References

71. Nurnberger JI, Blehar MC, Kaufmann CA, et al. Diagnostic interview for genetic studies: rationale, unique features, and training. Arch Gen Psychiatry 1994;51(11):849–59.

72. Lee SH, Ripke S, Neale BM, et al. Genetic relationship between five psychiatric disorders estimated from genome-wide SNPs. Nat Genet 2013;45(9):984–94.

73. plink. http://zzz.bwh.harvard.edu/plink/. Accessed 01 Feb 2020.
