# Supplementary material for: Gene-set Enrichment with Mathematical Biology (GEMB)
Source: Gigascience. 2020 Oct 9;9(10):giaa091. doi: 10.1093/gigascience/giaa091 (PMC7546080; doi:10.1093/gigascience/giaa091)
Supplement: giaa091_Response_to_Reviewer_Comments_Original_Submission [file giaa091_response_to_reviewer_comments_original_submission.pdf]

Author's Response To Reviewer Comments

Close

Thank you again for considering our revisions to our manuscript “Gene-Set Enrichment with Mathematical Biology (GEMB)” (GIGA-D-19-00048). We have updated the manuscript to address the latest comments from Reviewer #2. Changes are in boldface.

Response to Reviewer #2

1) Regarding novelty compared to other gene-set analysis (GSA), I believe the incorporation of math biology is a novel aspect indeed. As mentioned in the reply, "To account for this, some gene-set analyses incorporate information about the network structure of gene interactions. " Could you also cite the gene-set analysis methods and applications using network information and briefly explain what they have done, as opposed to the current method.

Response to 1) In the introduction, we updated the third paragraph to better describe other methods using network information and contrast these methods to the proposed method:

“Network-based methods, such as EnrichNet, GANPA, and LEGO, represent the network of functional interactions between genes as a network – a mathematical object made up of vertices (genes or proteins) and edges (connections). Based on features of the network, such as the number of connections a gene has with other genes, genes are given more or less weight in the gene-set test statistic. While these methods attempt to account for the functional non-equivalence of genes, they rely on mathematical properties of the network rather than biological mechanisms involved in the related biological function. The connections between genes might vary in strength or operate in a non-linear way. For example, a gene with many weak connections to other genes might carry equal or less downstream biological influence than a gene with few, strong connections to other genes. This discrepancy would be obscured by considering only the number or pattern of connections.”

In the discussion, we updated the sixth paragraph to provide more information on network-based analyses:

“There are a number of other methods like GEMB that try to incorporate information about gene function into gene-set analyses. Network-based methods represent genes as vertices and interactions between two genes as edges, resulting in a mathematical object known as a network. Properties of this network (e.g., their location in the network, number of connections to a gene) are used to adjust the weight given to a gene in a gene-set analysis. In a broad sense, genes might be important to a biological pathway if they are more connected to other genes. For example, GANPA and LEGO build a network based on gene co-expression, protein-protein interactions, and gene ontology. Based on this network, they weight each gene in the set of interest as a function of the number of connections to other genes in the set and other genes in the entire network. EnrichNet uses a random walk on the network to capture relationships between genes. Alternative ways to incorporate functional information about genes include Bayesian approaches that account for overlap between gene sets or approaches based on gene expression levels. A benefit of our method is that it is sufficiently general, such that weights could also be determined from network analysis, experiments or meta-analysis. Weights need only be non-negative and sum to one.”

2) On the use of quantitative GWAS significance rather than gene ranks, I would advise a more thorough discussion of the pros and cons of each (apart from sample size) in the discussion. (Eg. methods like MAGMA employs gene significance for their test)

Response to 2) Thank you for the suggestion. In the Discussion, we updated the third paragraph to further discuss gene ranks, with a particular focus on encouraging further study to evaluate gene-level measures:

“Popular gene-set tests typically start with gene-level measures of association between genes and the

phenotype based on statistics like fold change, t statistic, p value, likelihood ratio, regression coefficient, and correlation coefficient. We computed gene-level p values obtained from MAGMA and transformed to gene ranks. The choice of gene-level measure and any subsequent transformation is an important decision, since it can alter the findings from gene-set analyses in several ways. First, the chosen gene-level measure may yield better accuracy in finding significant gene sets depending on the genetic architecture of the disease of interest. A binary measure may be better than a gene rank for diseases with a few highly influential genes but worse for diseases with a large number of moderately influential genes. Second, gene-level measures differ in their statistical properties. For example, larger sample sizes may change p values without affecting gene ranks, and using a probit transformation of p values rather than p values directly, as in MAGMA, can yield a distribution closer to normal. Third, the choice of statistic could change the clinical relevance of the findings, as one finds in a single gene analysis when comparing fold change to a t statistic. Although we use gene ranks, ranks can be easily replaced by any gene-level measure of association if desired. It will be important for future studies to explore how different gene-level measures fare with the weighted gene-set test we propose here.”

3) It is encouraging that the authors have uploaded some weight profiles onto Github. I hope in the future the authors could upload more datasets or provide a more detailed tutorial on how to use such weights or derive weights on their own.

Response to 3) This is a great suggestion. As we continue to work on this method, we will make sure to add additional datasets and make our method accessible to a broad audience.

Close
