# Supplementary material for: Gene-set Enrichment with Mathematical Biology (GEMB)
Source: Gigascience. 2020 Oct 9;9(10):giaa091. doi: 10.1093/gigascience/giaa091 (PMC7546080; doi:10.1093/gigascience/giaa091)
Supplement: giaa091_Response_to_Reviewer_Comments_Revision_1 [file giaa091_response_to_reviewer_comments_revision_1.pdf]

Author's Response To Reviewer Comments

Close

We added the suggested links to the data availability section. We also registered the software on biotools and scicrunch.

Close
